# Natural product-modified BODIPY: design strategies, photophysical properties and biomedical applications

**DOI:** 10.1039/d6ra06055b

**Published:** 2026-08-18

**Authors:** Linlin Gai, Qianqian Wu, Weice Sun

**Affiliations:** a Medical Research Center, Weifang People's Hospital, Shandong Second Medical University Weifang 261000 China; b Department of Clinical Laboratory, Affiliated Hospital of Shandong Second Medical University Weifang 261000 China; c Vascular Surgery, Weifang Traditional Chinese Hospital Weifang 261000 China m19862676527@163.com

## Abstract

Natural products and boron-dipyrromethene (BODIPY) dyes represent highly complementary building blocks for the construction of multifunctional theranostic systems. The integration of the intrinsic biological activities and structural diversity of natural products with the excellent photophysical properties of BODIPY fluorophores has enabled the development of a wide range of hybrid platforms for biomedical imaging, phototherapy, drug delivery, and precision medicine. This review provides a comprehensive overview of recent advances in natural product-modified BODIPY systems, with a particular focus on their design strategies, structure–activity relationships, and biomedical applications. Finally, current challenges and future opportunities are highlighted to provide a unified framework for the rational design of next-generation natural product-modified BODIPY systems with enhanced biomedical potential.

## Abstract 2

Natural product-modified BODIPY systems integrate biological functions with tunable photophysical properties, enabling multifunctional platforms for imaging, phototherapy, drug delivery and precision theranostics.

## Graphical abstract

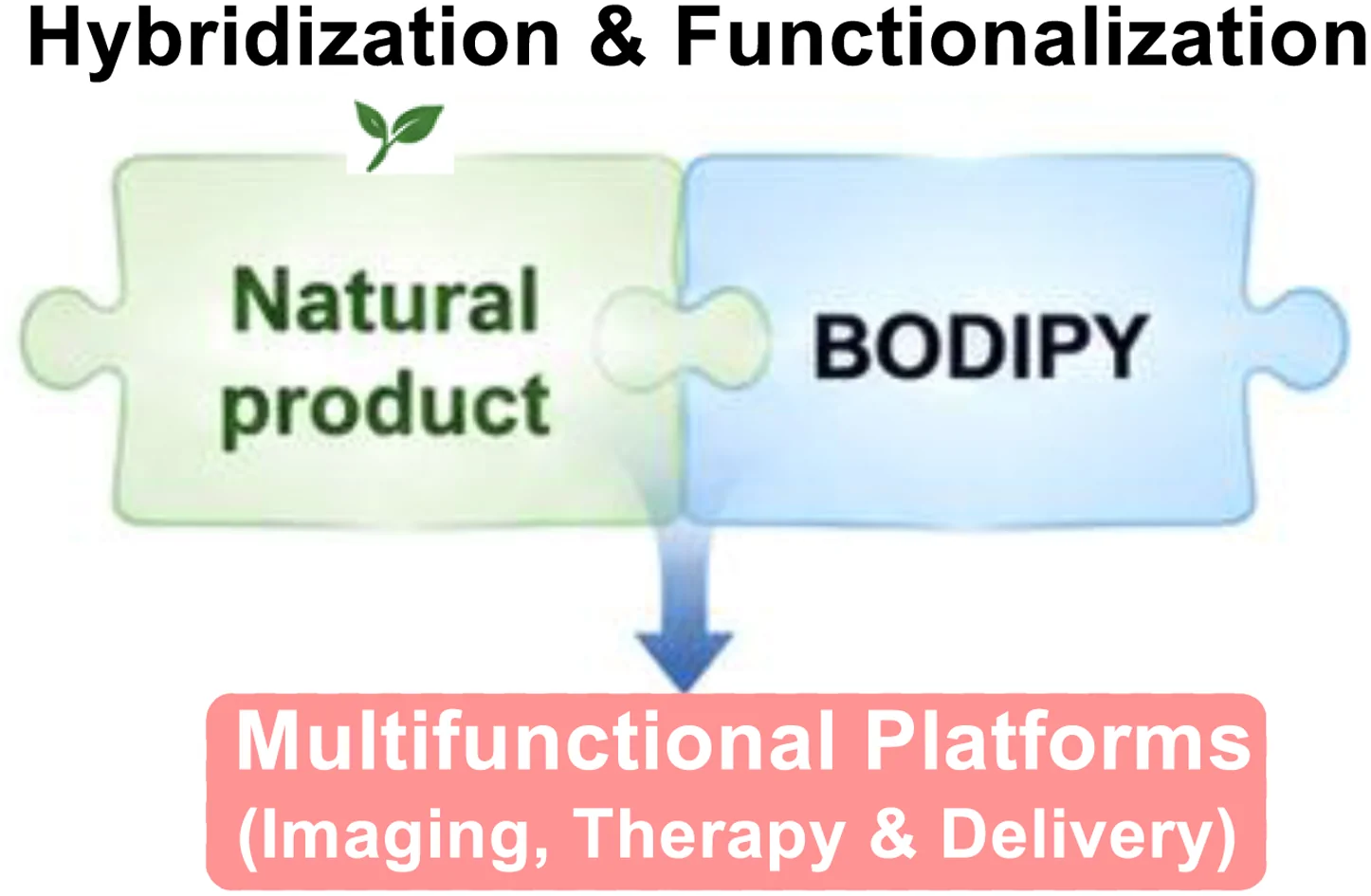

## 1 Introduction

Natural products have long served as an indispensable source of biologically active molecules for medicine, chemical biology, and materials science, offering unparalleled structural diversity, evolutionary optimization, and broad-spectrum activities that have yielded numerous clinically important agents.^1,2^ Beyond their direct therapeutic applications, many natural products possess intrinsic biocompatibility, receptor-recognition capability, organelle-targeting affinity, and favorable pharmacological profiles. These characteristics make them attractive molecular scaffolds for the construction of functional biomedical systems.^3–6^ However, the direct biomedical application of many natural products remains limited. Poor aqueous solubility, rapid metabolic degradation, low bioavailability, and insufficient target selectivity frequently compromise their therapeutic performance. In addition, most natural products lack suitable optical properties for real-time visualization and treatment monitoring. Consequently, strategies capable of preserving the biological functions of natural products while introducing imaging and therapeutic functionalities have attracted increasing attention.

Among various fluorescent scaffolds, boron-dipyrromethene (BODIPY) dyes have attracted exceptional attention owing to their favorable photophysical characteristics, including high molar absorptivity, sharp absorption and emission bands, excellent photostability, tunable spectral profiles, and versatile synthetic accessibility.^7–12^ Structural modification of the BODIPY core enables systematic regulation of fluorescence emission, triplet state generation, singlet oxygen production, and environmental responsiveness.^13–21^ These features have established BODIPY derivatives as valuable platforms for fluorescence imaging, sensing, photodynamic therapy, photothermal therapy, and theranostic applications.^16,22–31^

**Scheme 1:**
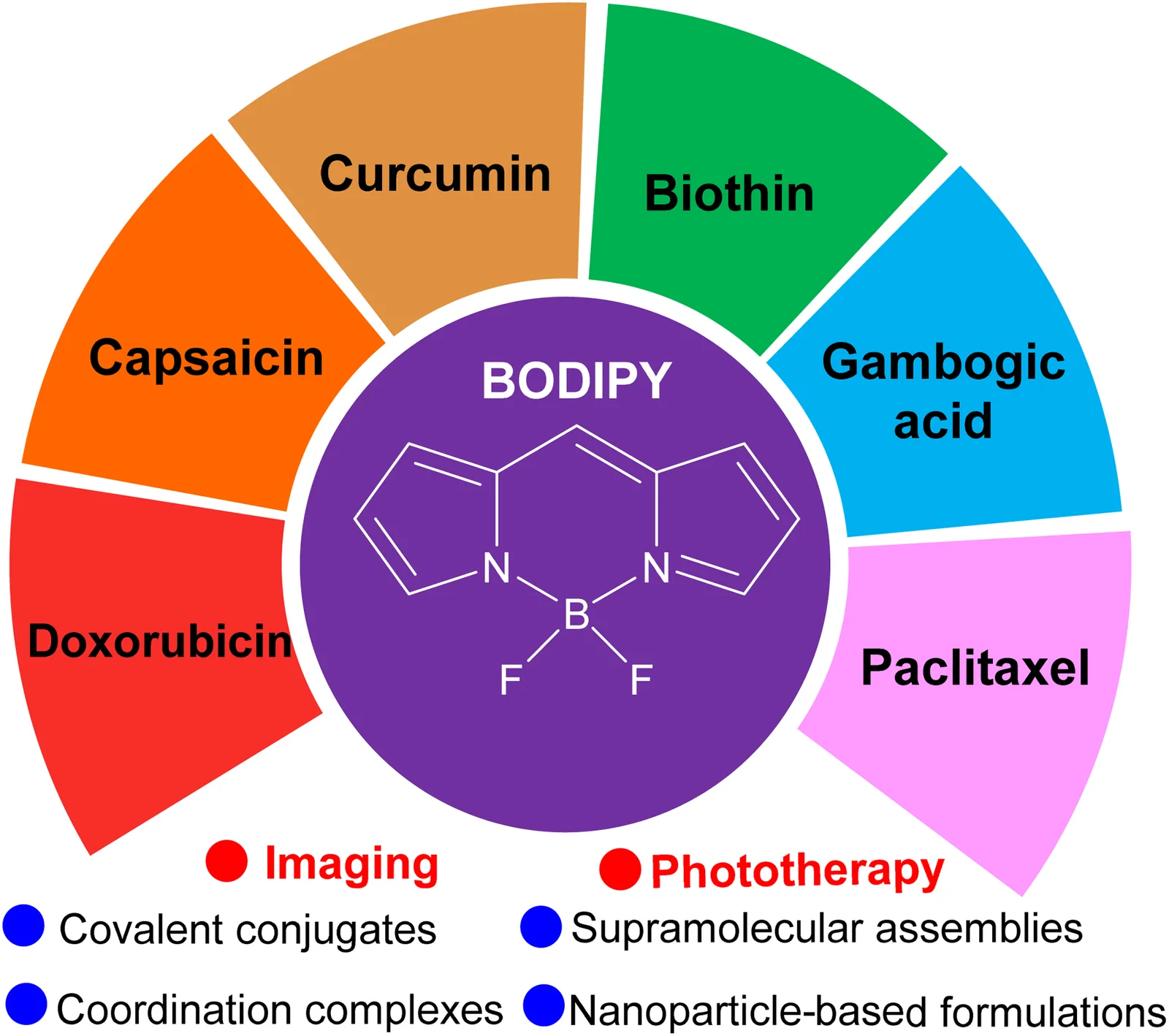
The classification of natural products modified BODIPYs.

The integration of natural products with BODIPY fluorophores extends beyond simple fluorescent labeling. Natural products contribute biologically validated targeting motifs, therapeutic activity, organelle affinity, or self-assembly capability, whereas BODIPY provides optical visualization, photo-responsiveness, and phototherapeutic functionality. The resulting hybrid systems often exhibit emergent properties, such as activatable imaging probes, stimuli-responsive prodrugs, self-reporting therapeutics, or multifunctional nanomedicines. Over the past decade, diverse natural products including cytotoxic agents (paclitaxel, gambogic acid), bioactive phytochemicals (curcumin, capsaicin), and endogenous biomolecules (biotin), have been incorporated into BODIPY architectures (Scheme 1). Recurring design principles include covalent conjugation for simultaneous imaging and therapy, stimuli-responsive linkers coupling fluorescence activation to drug release, carrier-free self-assembled nanostructures, and combining natural product targeting with BODIPY-mediated photodynamic activity.

Despite remarkable progress, several important challenges remain unresolved. The relationship between molecular structure, excited-state behavior, and biological performance is often insufficiently understood. Comprehensive photophysical characterization is frequently incomplete, limiting meaningful comparison between different studies. Furthermore, critical issues associated with pharmacokinetics, biodegradation, long-term safety, large-scale synthesis, and clinical translation remain largely unexplored. Several excellent reviews have summarized various aspects of BODIPY chemistry and its biomedical applications. Antina *et al.* provided a comprehensive overview of BODIPY conjugates with drugs, vitamins, hormones, steroids, lipids, and other bioactive molecules.^32^ Stanková *et al.* focused specifically on terpene and terpenoid based BODIPY conjugates, highlighting their chemical properties and biological applications.^33^ Mao *et al.* reviewed BODIPY based theranostic systems for cancer treatment,^20^ while Cheng *et al.* discussed BODIPY self- assembly strategies and their biomedical applications.^34^ Das *et al.* further summarized BODIPY based small molecules for disease diagnosis and therapeutic applications.^35^

Building upon these important contributions, this review provides a complementary perspective by adopting a natural product centered framework to systematically analyze BODIPY hybrids. Unlike previous reviews that primarily focus on BODIPY chemistry, specific biomedical applications, or individual subclasses of natural products, we examine six representative natural product families, including curcumin, gambogic acid, biotin, capsaicin, paclitaxel, and doxorubicin. Particular attention is given to how the intrinsic structural features of these molecules, such as metal binding motifs, targeting groups, pharmacophoric elements, and self-assembly capabilities, influence the design and performance of BODIPY based systems. Furthermore, we provide a comparative analysis of conjugation strategies, linker designs, BODIPY structural modifications, and biological outcomes across different natural product classes to identify broader design principles. A comprehensive summary table (Table 1) is also included to integrate key photophysical and biological parameters for the 28 representative compounds discussed. Finally, this review highlights translational challenges, including pharmacokinetic evaluation, toxicity assessment, synthetic reproducibility, scalability, and regulatory considerations, which remain insufficiently addressed in previous studies. Through this integrated and comparative analysis, we aim to provide a critical framework that complements existing literature and offers new insights into the rational design of natural product based BODIPY platforms.

**Table 1:** Key photophysical and biological parameters of natural product BODIPY hybrids 1–28 discussed in this review

Dye	Natural product	Key design feature	*λ*_abs_ (nm)	*λ*_em_ (nm)	*Φ*_F_	*Φ*_Δ_	IC_50_ light	IC_50_ dark	Photo-index (PI)
la	Curcumin	Pt(ii)–BODIPY conjugate	615	723	0.03	0.27	4.5 µM A549	56.2 µM A549	12.5
1b	Curcumin	Pt(ii)–BODIPY conjugate	635	845	0.01	0.40	1.3 µM A549	53.9 µM A549	41.5
2	Curcumin	Oxidovanadium(iv)–BODIPY	535	521	0.01	n.r.	2.7 µM (MCF-7)	99 µM (MCF-7)	36.6
3	Curcumin	Cu(ii)–BODIPY	501	512	0.12	n.r.	7.9 µM (HeLa)	29.1 µM (HeLa)	3.68
4	Curcumin	Cu(ii)–BODIPY	535	510	0.01	n.f.	3.8 µM (HeLa)	32.1 µM (HeLa)	8.4
5	Curcumin	Zn(ii)–BODIPY	445/501	506	0.54	0.18	1 µM (HeLa)	48.8 µM (HeLa)	48.8
6	Curcumin	Zn(ii)–BODIPY	543	506	0.02	0.73	25 nM (HeLa)	64.6 µM (HeLa)	2580
7	Gambogic acid	COF-encapsulated nanoplatform	407	n.r.	n.r.	n.r.	>95% viability (non-hypoxic)	n.r.	—
8	Gambogic acid	Covalent BODIPY conjugate	n.r.	n.r.	n.r.	n.r.	∼0.6 µM	n.r.	—
9a	Biotin	pH-switchable photosensitizer	693	760	n.r.	0.038 → 0.169 (pH 5.0)	22 nM (HeLa)	n.r.	—
10	Biotin	Lodinated Aza-BODIPY conjugate	648	703	0.027	0.72	3 µM (MDA-MB-231)	n.r.	—
11	Biotin	Click-conjugated BODIPY (PEG2)	534	551	0.032	0.33	18.7 nM (MDA-MB-231)	n.r. (negligible)	—
12	Biotin	Click-conjugated BODIPY (PEG4)	533	549	0.039	0.34	52 nM (MDA-MB-231)	n.r. (negligible)	—
13	Biotin	Dimeric BODIPY	497	515	0.03 (Water)	n.r.	—	—	—
							(Imaging)		
14	Biotin	Dimeric BODIPY (fluorogenic probe)	497	506	0.60 (MeOH)	n.r.	—	—	—
							(Imaging)		
15	Biotin	GSH-activatable BODIPY–ferrocene	638	648	Quenched	n.r.	—	—	—
							(Imaging)		
16	Biotin	Ru(ii)–terpyridyl BODIPY dyad	503	513	0.01	0.63	0.16 µM (HeLa)	>100 µM	625
17	Biotin	Pt(iv)–BODIPY prodrug	651	677	0.13	0.32	0.61 µM (A549)	>100 µM	>164
18	Biotin	Pt(iv)–Ru(ii)–BODIPY heterobimetallic	500	513	<0.01	0.64 (Type I)	∼0.022 µM (A549)	>100 µM	>4545
19	Capsaicin	Photo-caged PAVA–BODIPY probe	500	510–525	0.01	n.r.	—	—	—
							(Photo control)		
20	Capsaicin	Self-assembling amphiphilic BODIPY conjugate	495–501 (Monomer); 532 (aggregate)	503–509 (Organic); ∼620 (aqueous)	n.r.	n.r.	—	—	—
							(*In vivo* antitumor)		
21	Paclitaxel	BODIPY dimer (carrier-free NPs)	632/639	∼688	n.r.	n.r.	n.r.	n.r.	—
22	Paclitaxel	Redox-activated prodrug	∼500	n.r.	n.r.	n.r.	0.02 µM (HeLa)	Lower than light	—
23	Paclitaxel	Amphiphilic Pt-containing conjugate	501	540	0.54 (DMF/H_2_O)	n.r.	48 nM (A549)/60 nM (MCF-7)	n.r.	—
					0.01 (NPs)				
24	Paclitaxel	Photoactivatable super-resolution probe	498	517	0.002 (Initial)	n.r.	—	—	—
							(Imaging)		
25	Doxorubicin	Aza-BODIPY polymeric nanoplatform	480/652	570/730/830	n.r.	0.82	3.1 µg mL^−1^ (HeLa)	n.r.	—
26	Doxorubicin	Carrier-free self-assembled NPs	660 (BODIPY)	562, 588, 705	0.0233	0.71	—	—	—
							(*In vivo* efficacy)		
27	Doxorubicin	Covalent acid-labile polymeric prodrug	480/490	520	n.r.	n.r.	—	—	—
							(Multi-drug delivery)		
28	Doxorubicin	Supramolecular BODIPY macrocycle host–guest	511	541–547	0.053 (in H_2_O)	1.60 (160%)	n.r.	n.r.	—

## 2 Natural product modified BODIPY dyes

Before discussing representative examples, it is important to clarify the scope of natural-product-integrated BODIPY systems covered in this review. Here, we define these systems as molecular or supramolecular constructs in which a natural product (or a structurally related derivative) is deliberately integrated with a BODIPY fluorophore through covalent, coordination, or non-covalent interactions to enable imaging, therapeutic, or theranostic applications. Based on the mode of integration and the level of structural organization, these systems can be broadly classified into four categories:

(i) Covalent conjugates, in which natural products and BODIPY fluorophores are connected through stable covalent linkages, including amide, ester, triazole, or ether bonds.^33,36^ This approach represents the most straightforward and widely adopted strategy, providing precise control over molecular architecture, stoichiometry, and linker chemistry while enabling systematic modulation of the physicochemical and biological properties of both components.

(ii) Coordination complexes, in which metal centers serve as bridging units to integrate natural-product ligands and BODIPY chromophores, either through direct coordination or covalent incorporation of metal-binding motifs.^37,38^ Representative examples include curcumin-based metal complexes utilizing β-diketonate coordination and biotin-directed systems incorporating Pt(iv) or Ru(ii) centers for combined targeting, therapeutic, and imaging functions.

(iii) Supramolecular assemblies, in which natural-product-BODIPY conjugates undergo spontaneous organization through non-covalent interactions, such as π–π stacking, hydrogen bonding, and hydrophobic interactions, generating ordered nanoarchitectures without extensive reliance on external carriers.^39,40^ These systems include carrier-free nanomedicines and host–guest assemblies.

(iv) Nanostructured delivery systems, in which natural products, BODIPY conjugates, or both are incorporated into engineered nanocarriers, including polymeric nanoparticles, covalent organic frameworks (COFs), and lipid-based platforms. Compared with molecular conjugates, these systems primarily focus on improving solubility, pharmacokinetics, biodistribution, and therapeutic efficacy.^41,42^

In the following sections, representative examples from each category are discussed, with emphasis on molecular design strategies, structure–property relationships, and biological outcomes. Hybrid systems that combine multiple design principles are also highlighted, reflecting the increasing convergence of molecular engineering, supramolecular chemistry, and nanomedicine.

### 2.1 Curcumin modified BODIPY dyes

Curcumin is a naturally occurring polyphenolic compound isolated from the rhizomes of *Curcuma longa*. Structurally, it consists of two *o*-methoxy phenolic rings linked by an α,β-unsaturated β-diketone moiety, forming an extended conjugated system that exhibits keto–enol tautomerism.^43^ This unique structure endows curcumin with potent antioxidant activity and a broad spectrum of pharmacological properties, including anti-inflammatory, antimicrobial, neuroprotective, and anticancer effects.^44^ Extensive studies have demonstrated that curcumin exerts its antitumor activity through modulation of multiple signaling pathways, such as Nuclear factor kappa-B (NF-κB), signal transducer and activator of transcription 3 (STAT3), phosphoinositide 3-kinase/protein kinase B/mammalian target of rapamycin (PI3K/Akt/mTOR), and mitogen-activated protein kinase (MAPK), leading to inhibition of cancer cell proliferation, induction of apoptosis, suppression of angiogenesis, and prevention of metastasis.^45,46^ Curcumin can also regulate oxidative stress and inflammatory responses within the tumor microenvironment, further contributing to its anticancer efficacy.^46^ Despite these advantages, clinical translation of curcumin remains challenging. Poor aqueous solubility, rapid metabolic clearance, low systemic bioavailability, and limited chemical stability significantly restrict its therapeutic efficacy. Nevertheless, the presence of phenolic hydroxyl groups and a chemically accessible β-diketone motif provides versatile handles for structural modification. Consequently, curcumin has emerged as an attractive natural-product scaffold for the construction of multifunctional theranostic systems. Importantly, the extended π-conjugation and metal-chelating ability of curcumin render it particularly compatible with BODIPY-based molecular engineering strategies, enabling synergistic structure–function integration that is difficult to achieve using either component alone.

**Fig. 1:**
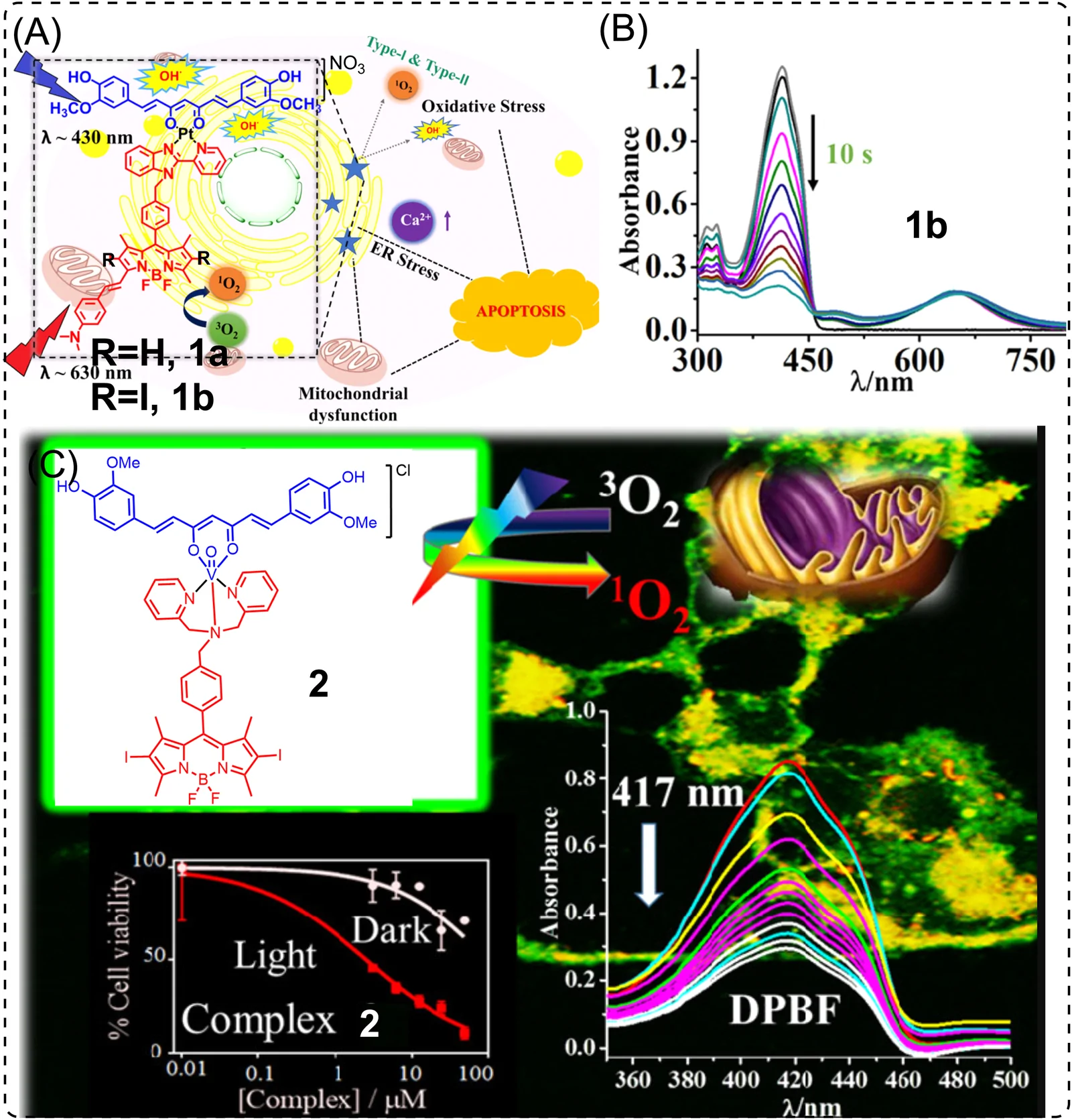
(A) Structure of dye 1a–1b and Schematic illustration of photodynamic antitumor therapy based on natural product-BODIPY hybrids 1a–1b. (B) The spectral changes of DPBF (40 µM) at 415 nm were monitored over time in air-saturated DMF under red light irradiation (*λ* = 642 nm) in the presence of complex 1b (reproduced with permission.^47^ Copyright 2022, *American Chemical Society*). (C) Structure of dye 2 and schematic diagram of photodynamic antitumor effects (reproduced with permission.^50^ Copyright 2017, *American Chemical Society*).

Among the earliest examples, BODIPY-tagged platinum(ii)–curcumin complexes (compounds 1a and 1b) were constructed through coordination of curcumin in its β-diketonate enolate form to a square-planar Pt(ii) center together with a pyridyl-benzimidazole ligand covalently linked to mono- or diiodo-styryl BODIPY chromophores (Fig. 1A).^47^ The complexes exhibit characteristic BODIPY-centered absorption maxima at 615 nm (1a, *ε* = 4.1 × 10^4^ M^−1^ cm^−1^) and 635 nm (1b, *ε* = 3.4 × 10^4^ M^−1^ cm^−1^), accompanied by curcumin-derived bands at 445 and 492 nm, respectively. 1a displays fluorescence emission at 723 nm with a quantum yield of 0.03, whereas 1b emits at 845 nm with a markedly lower *Φ*_F_ of 0.01 due to enhanced heavy-atom-induced intersystem crossing (ISC). The pronounced bathochromic shift into the therapeutic window is particularly significant because it improves tissue penetration relative to conventional visible-light-absorbing BODIPY systems. This hybrid architecture combines the anticancer, anti-inflammatory, and endoplasmic reticulum (ER)-affinitive characteristics of curcumin with the strong red-light absorption, photostability, and ROS-generation capability of BODIPY–Pt(ii) systems. Functionally, these complexes act as ER-targeted photodynamic therapeutic agents, with 1b exhibiting efficient singlet oxygen generation (*Φ*_Δ_ = 0.40) (Fig. 1B)^13,48,49^ and potent photocytotoxicity under red-light irradiation (IC_50_ = 1.3–6.9 µM), while 1a simultaneously enables intracellular imaging through its red fluorescence. These findings demonstrate how subtle structural modifications can shift the balance between imaging performance and photodynamic efficacy.

Building upon this concept, replacement of Pt(ii) with redox-active transition metals has emerged as an alternative strategy for simultaneously enhancing curcumin stability and introducing new therapeutic mechanisms. Curcumin–oxidovanadium(iv)–BODIPY conjugates are ternary coordination assemblies in which curcumin is stabilized through O,O-chelation in its monoanionic enolic form to an oxidovanadium(iv) center, while diiodo-BODIPY chromophores serve as pendant photoactive units (Fig. 1C).^50^ The conjugates 2 display absorption maxima at 535 nm (*ε* = 30 285 M^−1^ cm^−1^), fluorescence emissions at 521 nm, and fluorescence quantum yields of 0.01, respectively. The marked reduction in fluorescence efficiency in the iodinated analogue reflects effective intersystem crossing and enhanced triplet-state formation. Compared with the Pt(ii)-based systems, these complexes operate in a shorter wavelength regime but benefit from the intrinsic redox activity of vanadium, which may contribute additional oxidative stress pathways in cancer cells. Consequently, therapeutic efficacy is derived not only from photoinduced ROS generation but also from the biological activity of the metal center itself. Functionally, the complexes preferentially accumulate in mitochondria and act as photodynamic anticancer agents. The diiodo-BODIPY conjugate 2 exhibits potent visible-light-induced cytotoxicity with IC_50_ values of 2.7 µM in michigan cancer foundation-7 (MCF-7) cells and 2.5 µM in HeLa cells, while remaining markedly less toxic in the dark (∼99 and 55 µM, respectively), and generates reactive oxygen species (ROS) through combined singlet-oxygen and hydroxyl–radical pathways. These findings underscore the importance of integrating organelle targeting with controlled ROS production, as mitochondrial localization significantly amplifies photodynamic damage by acting directly on cellular bioenergetic pathways.

**Fig. 2:**
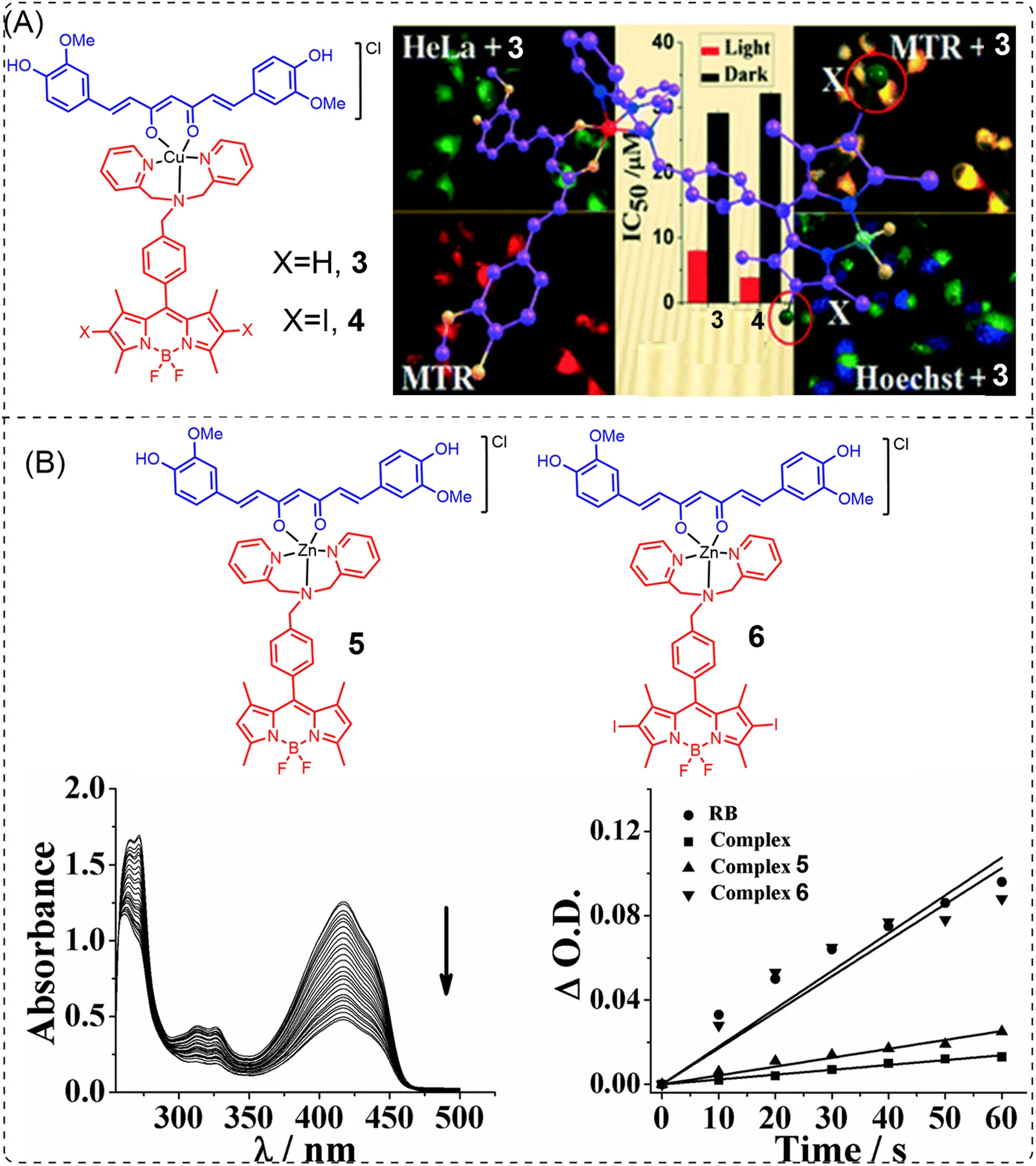
(A) Chemical structures of complexes 3–4 and schematic diagram of photodynamic anti-tumor effects (reproduced with permission.^51^ Copyright 2015, *Royal Society of Chemistry*). (B) Chemical structures of complexes 5–6. Photodynamic-related experimental data and corresponding schematic representation (reproduced with permission.^52^ Copyright 2021, Elsevier).

A related design philosophy has been applied to copper(ii)-based systems. BODIPY-appended copper(ii)–curcumin complexes (3–4) are formed through coordination of curcumin and a tridentate BODIPY-functionalized dipicolylamine ligand to a Cu(ii) center, generating a distorted square-pyramidal CuN_3_O_2_ core (Fig. 2A).^51^ The complexes display absorption maxima at 501(for 3) and 535 nm (for 4), fluorescence emissions at 512 (for 3) and 510 nm (for 4), and fluorescence quantum yields of 0.12 (for 3) and 0.01 (for 4), respectively. As observed for the vanadium analogues, incorporation of iodine atoms dramatically enhances triplet-state formation at the expense of fluorescence efficiency. The close similarity in photophysical behavior between the copper and vanadium systems suggests that the BODIPY chromophore remains the dominant determinant of excited-state properties, whereas the coordinated metal primarily influences biological performance and molecular stability. Biologically, the complexes 3–4 localize preferentially within mitochondria and function as photodynamic agents in HeLa cells. The diiodo-BODIPY derivative 4 exhibits potent visible-light photocytotoxicity (IC_50_ = 3.8 ± 0.2 µM) while remaining substantially less toxic in the dark (IC_50_ = 32.1 ± 0.4 µM), with efficacy comparable to that of Photofrin. Such results demonstrate that rational metal selection can provide a practical route to improving the pharmacological robustness of curcumin without compromising the optical performance of the BODIPY component.

In contrast to redox-active transition metals, Zn(ii) offers a diamagnetic and biologically benign coordination environment. Ternary zinc(ii) complexes feature a bioessential Zn(ii) center coordinated by an O,O-donor curcumin ligand and a BODIPY-functionalized tridentate dipicolylamine ligand, affording a stable distorted trigonal bipyramidal five-coordinate geometry. Curcumin–Zn(ii)–BODIPY complexes (5–6) were therefore developed as imaging-oriented theranostic systems (Fig. 2B).^52^ Under simulated physiological conditions (1% DMSO–DPBS, pH 7.4), the heavy-atom-free emissive conjugate (complex 5) exhibits distinct photophysical features, displaying a strong curcumin-centered π–π* absorption at 445 nm, a prominent BODIPY HOMO–LUMO transition at 501 nm, and an emission maximum at 506 nm with a high fluorescence quantum yield of 0.54; conversely, the diiodinated photosensitizing analogue (complex 6) undergoes a bathochromic shift in the BODIPY absorption to 543 nm and a heavy-atom-induced quenching of fluorescence of 0.02, which drastically promotes the singlet oxygen generation quantum yield from 0.18 to 0.73. The substantially higher fluorescence efficiency relative to the corresponding heavy-atom-containing systems highlights the trade-off between imaging brightness and photodynamic activity that frequently governs photosensitizer design. In this system, the zinc center serves a dual role: it stabilizes the otherwise hydrolysis-prone β-diketone framework of curcumin and enhances aqueous compatibility, while the BODIPY chromophore provides the strong light-harvesting capability required for effective photodynamic action. This division of labor between metal coordination and dye photophysics allows the two components to operate synergistically without mutual interference. Consequently, complex 6 enable efficient intracellular imaging and visible-light-triggered photodynamic therapy (PDT), achieving sub-micromolar photocytotoxicity with IC_50_ values as low as 25–55 nM.

Comparison of these curcumin-based coordination systems reveals several notable structure–property–function relationships. First, iodination of the BODIPY core consistently alters the excited-state dynamics by promoting intersystem crossing, thereby shifting the photophysical balance from fluorescence emission toward singlet-oxygen generation. This trend is evident from the comparisons of 1a/1b (*Φ*_F_: 0.03 → 0.01; *Φ*_Δ_: 0.27→0.40), 3/4 (*Φ*_F_: 0.12 → 0.01), and 5/6 (*Φ*_F_: 0.54 → 0.02; *Φ*_Δ_: 0.18 → 0.73), suggesting that the heavy-atom effect provides an effective strategy for enhancing photodynamic activity in these systems. Second, the coordinated metal center plays a decisive role in regulating both physicochemical properties and biological responses. Pt(ii)– and Cu(ii)-containing complexes generally exhibit enhanced photocytotoxicity, whereas Zn(ii)-based systems often retain higher fluorescence efficiency and therefore provide improved imaging capability. Third, redox-active metal ions, such as V(iv) and Cu(ii), may introduce additional oxidative pathways that synergize with BODIPY-mediated photodynamic processes, while Zn(ii) primarily contributes to structural stabilization and may improve the integrity of the curcumin β-diketone coordination motif. Collectively, these examples highlight that metal selection is a key design parameter rather than merely a synthetic consideration, and should be carefully tailored to achieve the desired balance between fluorescence imaging and therapeutic efficacy.

### 2.2 Gambogic acid modified BODIPY

Gambogic acid (GA) is a naturally occurring caged xanthone isolated from *Garcinia hanburyi* resin.^53^ It features a rigid, three-dimensional cage architecture decorated with isoprenyl side chains, α,β-unsaturated carbonyl moieties, and a free carboxyl group. This unique structural combination imparts pronounced hydrophobicity and contributes to its broad-spectrum anticancer activity.^54^ GA has emerged as a privileged natural-product scaffold, exhibiting efficacy against multiple cancer types, including breast, lung, liver, and pancreatic cancers, while also demonstrating the ability to circumvent multidrug resistance.^55^ Mechanistically, GA interacts with a diverse range of molecular targets, including transferrin receptor 1 (TfR1), heat shock protein 90 (Hsp90), nuclear factor kappa-B (NF-κB), and vascular endothelial growth factor receptor 2 (VEGFR2), resulting in ROS-mediated mitochondrial dysfunction, caspase-dependent apoptosis, and inhibition of angiogenesis and metastasis.^55,56^ From a medicinal chemistry perspective, the carboxyl group of GA provides a synthetically accessible site for structural modification without extensively perturbing the pharmacophore. More importantly, the inherent mitochondrial tropism of GA offers a unique opportunity to exploit subcellular targeting in theranostic probe design, a feature that is difficult to achieve using synthetic fluorophores alone.

**Fig. 3:**
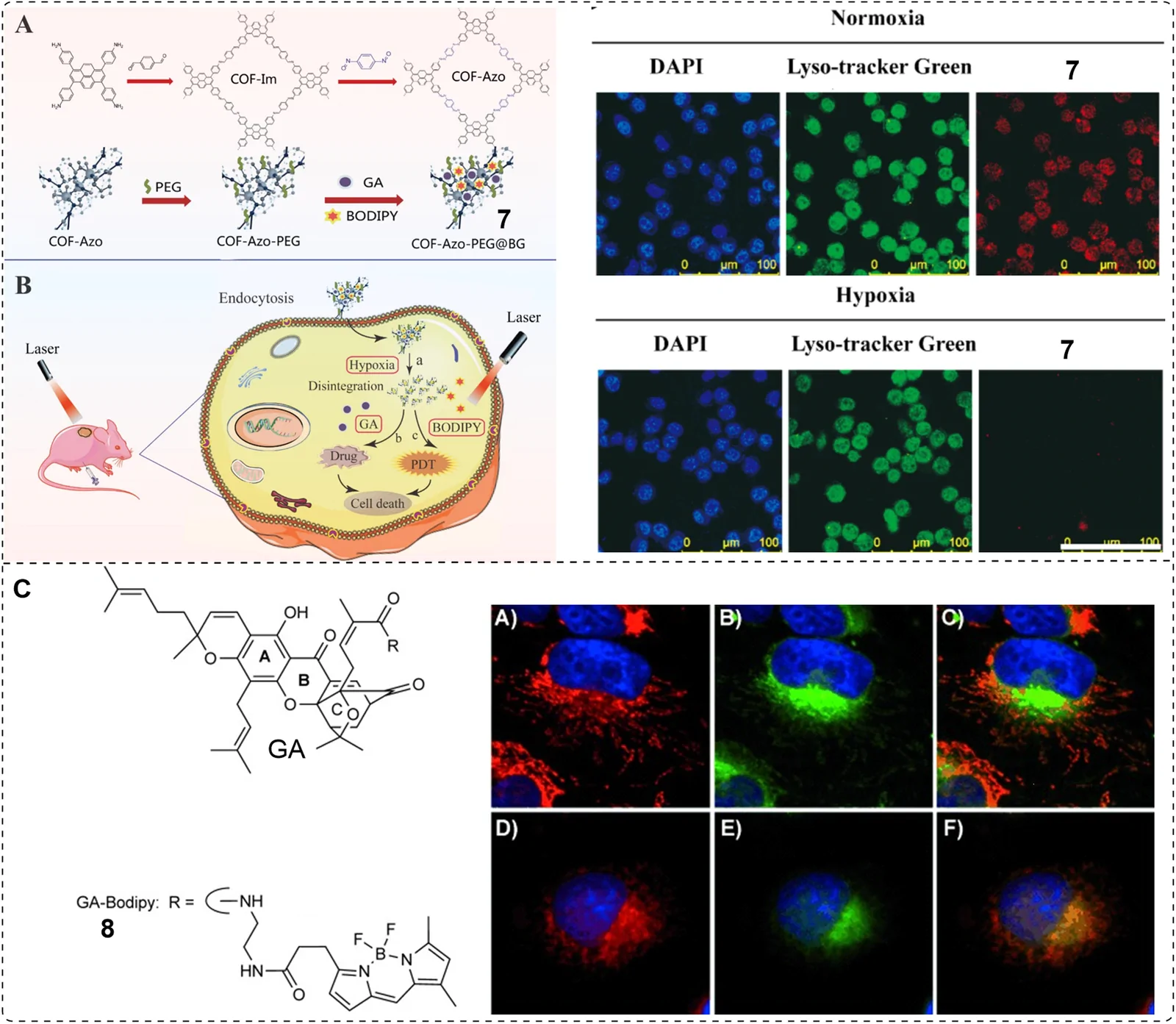
(A) Gambogic acid nanomedicine delivery platform. (B) Synthesis of the gambogic acid loaded COF nanomedicine 7. Mechanism of action of the nanomedicine for treating lung cancer. Three-channel fluorescence imaging of A549 cells under normoxia and hypoxia (reproduced with permission.^57^ Copyright 2025, Elsevier). (C) Chemical structures of gambogic acid (GA) and GA-BODIPY 8. GA can displace GA-BODIPY 8 from mitochondria. HeLa cells treated with 1 µM GA-BODIPY 8 for 30 min followed by washing. (A) and (D) Show mitochondria (red, anti-Tom20 antibody), while (B) and (E) show GA-BODIPY (green). (C) and (F) Present the merged images, with nuclei stained blue (reproduced with permission.^58^ Copyright 2012, John Wiley & Sons).

Beyond direct covalent conjugation, GA has also been incorporated into multifunctional supramolecular delivery systems designed to address its poor aqueous solubility, rapid systemic clearance, and dose-limiting toxicity. In one representative example, hypoxia-responsive nanomedicines were constructed the non-covalent encapsulation of the natural product gambogic acid and a synthetic boron-dipyrromethene fluorescent photosensitizer within the porous architecture of an azobenzene-functionalized covalent organic framework (COF) (Fig. 3A and B).^57^ The resulting system (7) integrates chemotherapy, photodynamic therapy, and microenvironment-responsive drug release within a single supramolecular architecture. The nanoplatform exhibits a characteristic absorption band centered at 407 nm and undergoes rapid fluorescence quenching under hypoxic conditions following reductive cleavage of the azobenzene linker. Importantly, the fluorescence response is directly coupled to structural disassembly of the framework. As a result, fluorescence changes provide a real-time optical readout of nanocarrier degradation and payload release. Such a design transforms the BODIPY component from a passive fluorescent label into an active reporter of therapeutic activation. The architecture of system 7 illustrates a high degree of functional complementarity. The porous COF framework improves the formulation of highly hydrophobic GA and enhances systemic biocompatibility. Simultaneously, azobenzene units confer selective responsiveness toward the reductive and hypoxic tumor microenvironment. This hierarchical design enables spatially controlled activation of both chemotherapy and photodynamic therapy while minimizing premature drug leakage during circulation. Importantly, cell viability of 7 remained above 95% under non-hypoxic conditions, highlighting the low off-target toxicity of the platform. These results highlight the value of integrating natural-product pharmacology with stimulus-responsive materials chemistry. Rather than serving merely as a drug cargo, GA actively participates in the overall therapeutic mechanism, thereby contributing to the multifunctionality of the nanomedicine.

In contrast to supramolecular delivery systems, direct molecular fusion of GA with BODIPY fluorophores provides an opportunity to investigate the intracellular behavior of the natural product itself. Such an approach is particularly valuable because the molecular targets and subcellular trafficking pathways of many natural products remain incompletely understood. GA-BODIPY 8 is a fluorescent conjugate in which gambogic acid is covalently linked to a BODIPY fluorophore, combining the highly emissive boron-dipyrromethene scaffold with the biologically active caged xanthone framework (Fig. 3C).^58^ The molecular design successfully integrates the favorable optical properties of BODIPY with the mitochondria-targeting and apoptosis-inducing characteristics of GA while largely preserving biological potency (IC_50_ ≈ 0.6 µM for GA-BODIPY *versus* 0.4 µM for GA). This relatively small loss of activity suggests that the selected conjugation site does not substantially interfere with the key pharmacophoric elements responsible for target engagement. Functionally, GA-BODIPY enabled direct visualization of the intracellular distribution of GA and exhibited strong mitochondrial colocalization, with a Pearson correlation coefficient of approximately 0.7 and a Manders' M1 coefficient of 0.94. Furthermore, competitive displacement by excess unlabeled GA demonstrated that mitochondrial localization was not merely a consequence of lipophilic accumulation but was associated with the underlying recognition properties of the parent natural product. Such visualization-driven approaches therefore represent a significant departure from conventional biochemical assays, as they directly link the subcellular distribution of gambogic acid to its site-of-action relationships, providing a dynamic and context-rich understanding of its pharmacology that is difficult to obtain through endpoint measurements alone.

Comparison of these two GA-based systems highlights the complementary advantages of covalent conjugation and supramolecular assembly strategies. The covalent conjugate 8 largely retains the biological activity of GA while enabling direct visualization of intracellular localization and trafficking, making it particularly valuable for mechanistic investigations. In contrast, the supramolecular nanoplatform 7 emphasizes enhanced drug loading capacity and microenvironment-responsive combination therapy, integrating chemotherapy and PDT within a dynamically assembled architecture. Although these two approaches differ in their levels of molecular definition and functional organization, they provide distinct advantages: covalent systems offer precise control over molecular interactions and biological tracking, whereas supramolecular platforms facilitate multifunctional delivery and improved therapeutic integration. Together, these examples illustrate how different structural strategies can be tailored to address specific requirements in natural-product-based theranostic design.

### 2.3 Biotin modified BODIPY

Biotin (vitamin B7 or H) is a water-soluble vitamin characterized by a ureido ring fused to a tetrahydrothiophene ring and a valeric acid side chain.^59^ As an essential cofactor for carboxylases, it participates in fundamental metabolic pathways, including fatty acid biosynthesis, gluconeogenesis, and amino acid metabolism. Unlike many natural products employed in cancer therapy, biotin itself possesses negligible intrinsic cytotoxicity. However, its value in molecular design arises from its ability to exploit the elevated metabolic requirements of rapidly proliferating cancer cells. Numerous tumor types, including breast, ovarian, lung, and cervical cancers, overexpress sodium-dependent multivitamin transporters (SMVTs) and biotin receptors, thereby providing a biologically validated route for selective cellular uptake.^60,61^ From a synthetic perspective, the terminal carboxyl group offers a convenient site for conjugation while generally preserving receptor recognition. Consequently, biotin has emerged as one of the most widely utilized endogenous targeting ligands in BODIPY-based theranostic systems. Unlike cytotoxic natural products, biotin serves primarily as a delivery vector,^62–64^ and its integration with BODIPY has given rise to several distinct design paradigms.

**Fig. 4:**
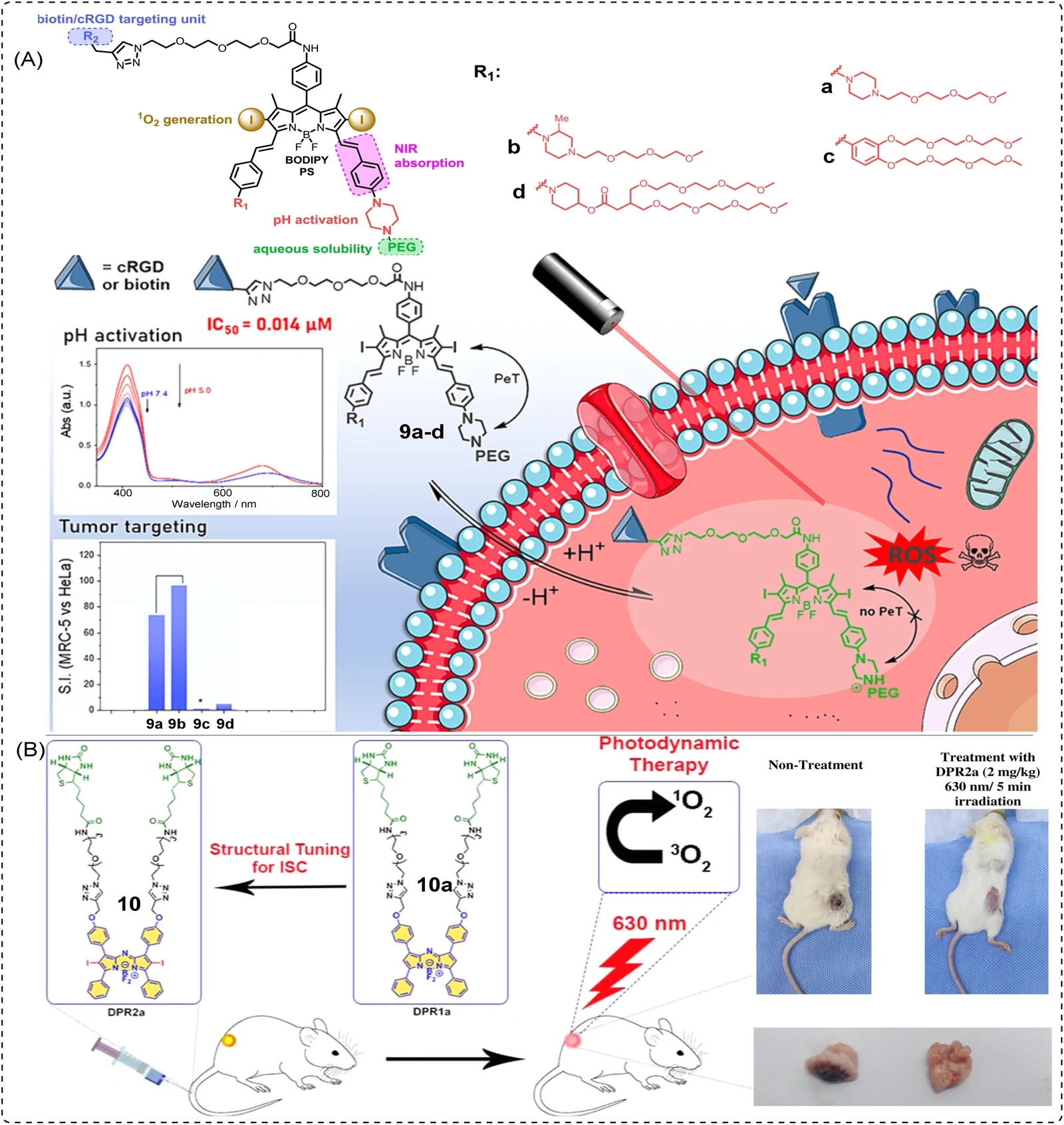
(A) General scaffold of pH-activatable and biotin targeted BODIPY 9a–9d. pH dependent activation of singlet oxygen generation indicated by DPBF method for compound 9b (reproduced with permission.^65^ Copyright 2024, Elsevier). (B) Structure of 10. PDT against MDA-MB-231 cancer cells using 10 in NOD/SCID mice (reproduced with permission.^66^ Copyright 2023, *American Chemical Society*).

Building upon simple receptor targeting, subsequent designs have increasingly incorporated stimuli-responsive mechanisms to further improve tumor specificity. Rather than relying solely on differential cellular uptake, these systems exploit characteristic features of the tumor microenvironment to regulate photodynamic activity. An illustrative example is the biotinylated pH-switchable photosensitizer 9a–9d, in which biotin is linked to a diiodinated distyryl BODIPY containing piperazinyl proton-responsive groups *via* a copper(i)-catalyzed azide–alkyne cycloaddition click reaction (Fig. 4A).^65^ Compound 9a exhibits absorption at 693 nm (62 000 M^−1^ cm^−1^) and remains photophysically suppressed at physiological pH owing to photoinduced electron transfer (PeT). Under acidic conditions, protonation inhibits PeT, leading to activation of fluorescence and ROS generation. Consequently, the singlet oxygen quantum yield of 9a increases from 0.038 at pH 7.4 to 0.169 at pH 5.0, accompanied by remarkable photocytotoxicity against HeLa cells (IC_50_ = 22 nM). Ultimately, the integration of a biotin-guided delivery vector with a pH-responsive, NIR-absorbing BODIPY photosensitizer represents a highly precise, dual-targeted therapeutic strategy capable of maximizing cancer cell destruction while avoiding off-target toxicity.

A similar design philosophy is evident in iodinated aza-boron-dipyrromethene (Aza-BODIPY) and BODIPY–biotin conjugates such as 10, in which atural product biotin is covalently coupled *via* a copper(i)-catalyzed azide–alkyne cycloaddition click reaction through an azide-functionalized PEG spacer to the core of an aza-boron-dipyrromethene (aza-BODIPY) scaffold modified with heavy iodine atoms (Fig. 4B).^66^ In these systems, heavy iodine substitution intentionally suppresses fluorescence in favor of efficient triplet-state formation. 10 exhibits absorption at 648 nm, emission at 703 nm, and a low fluorescence quantum yield of 0.027, while maintaining a triplet-state quantum yield of 75% and a singlet oxygen generation efficiency of 72%. In application, the bioconjugate demonstrates outstanding targeted efficacy, achieving a half-maximal inhibitory concentration IC_50_ of 3 µM in biotin-receptor-positive human triple-negative breast cancer MDA-MB-231 breast cancer cells compared to 30 µM in normal MCF cells, while *in vivo* administration of a 2 mg kg^−1^ dose to tumor-bearing non-obese diabetic/severe combined immunodeficient (NOD/SCID) mice under 630 nm laser irradiation drives complete tumor disappearance within 33 days.

**Fig. 5:**
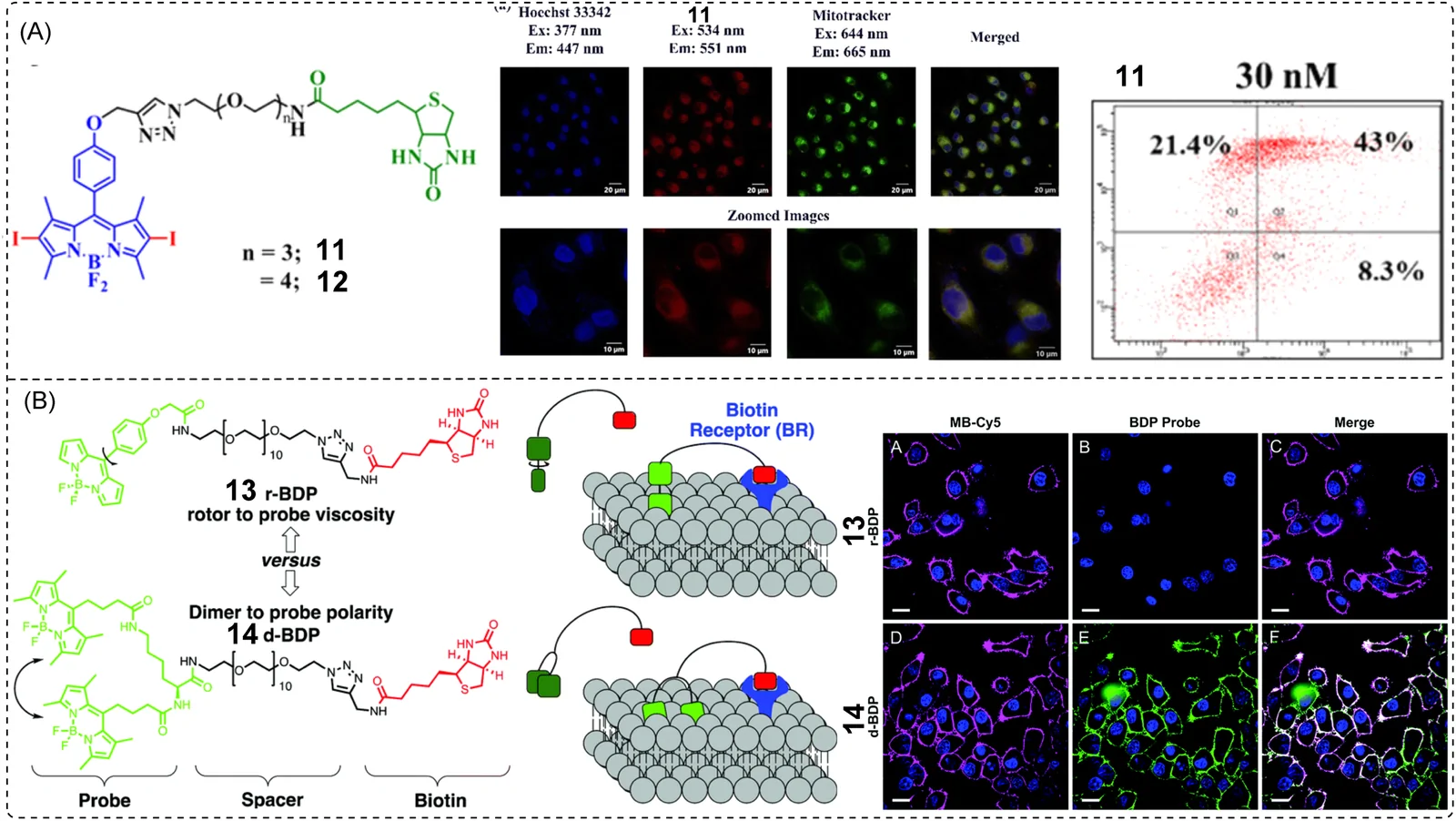
(A) Structures of BODIPY–biotin conjugates 11–12. Cellular localization and flow cytometric analysis of 11–12 in MDA-MB-231 breast cancer cells after 1 h of incubation (reproduced with permission.^67^ Copyright 2023, *Royal Society of Chemistry*). (B) Two fluorogenic probes for biotin receptors based on a BODIPY molecular rotor (r-BDP 13) and dimerized BODIPY (d-BDP 14). Confocal spinning disk imaging of KB cells in the presence of BODIPY probes 13–14 (reproduced with permission.^68^ Copyright 2021, *Royal Society of Chemistry*).

The conjugation of BODIPY with the natural product biotin is elegantly achieved *via* covalent attachment through copper-catalyzed azide–alkyne cycloaddition (click chemistry: this reaction proceeds through a copper(i)-catalyzed 1,3-dipolar cycloaddition between an organic azide and a terminal alkyne to form a 1,4-disubstituted 1,2,3-triazole linkage), anchoring a diiodinated BODIPY core to the terminal biotin moiety through triazole linkages and flexible poly(ethylene glycol) spacers PEG_3_ or PEG_4_ to construct amphiphilic theranostic hybrids designated as 11 and 12 (Fig. 5A).^67^ In methanol, the conjugates 11 and 12 exhibit prominent photophysical and photochemical attributes, displaying absorption maxima at 534 and 533 nm with exceptionally high molar absorptivities, respectively, alongside corresponding emission maxima at 551 and 549 nm; owing to heavy-atom-mediated intersystem crossing induced by the iodine substituents, their fluorescence quantum yields are drastically attenuated to 0.032 and 0.039, thereby driving efficient singlet oxygen generation quantum yields of 0.33 and 0.34. This structural architecture uniquely orchestrates the functionalities of its constituent modules: the BODIPY core delivers a compact molecular size and excellent light-harvesting capability, bypassing the synthetic complexity and low molar absorptivity characteristic of traditional macrocyclic porphyrinoids, while optimizing triplet-state population *via* strategic diiodination; simultaneously, the biotin moiety preserves its prominent specificity and strong affinity for biotin receptors (BRs) overexpressed on malignant cell membranes, such that their spatial link *via* a hydrophilic PEG tether fine-tunes the lipophilic–hydrophilic balance and harmoniously merges exceptional photo-responsiveness with tumor-selective homing capabilities. *In vitro* biomedical applications, these conjugates display remarkable theranostic utility by enabling rapid, exclusive localization within the mitochondria of cancer cells within 1 h; specifically, 11 exerts potent visible-light-induced photocytotoxicity against MDA-MB-231 breast cancer cells with a sub-micromolar IC_50_ value of 18.7 nM (12, 52.2 nM) alongside negligible dark cytotoxicity, primarily by driving a massive surge in intracellular ROS population (from 1.6% in the light control to 41.6% at 20 nM) to elicit a 21.8% G2/M phase cell-cycle arrest and trigger intrinsic apoptotic pathways. Overall, the orchestrated click metallo-free assembly of a high-extinction diiodinated BODIPY with a receptor-targeted biotin ligand effectively transcends the selectivity constraints of classical photodynamic agents, establishing a robust design paradigm for stable, mitochondrion-targeted, and visible-light-activated precision oncological platforms.

While the preceding systems focus on photodynamic therapy, biotin has also been widely employed in fluorogenic imaging probes, where target-to-background contrast rather than ROS generation becomes the primary design criterion. For example, a biotin-functionalized dimeric BODIPY (14) was constructed through the natural product biotin is conjugated to a dimeric BODIPY core *via* a PEG12 spacer and lysine linker, enabling biological targeting (Fig. 5B).^68^ Evaluated in methanol to simulate an organic membrane environment, this 14 construct features a maximum absorption wavelength of 497 nm, a molar absorption coefficient of 160 000 M^−1^ cm^−1^, a fluorescence emission maximum of 506 nm, and a fluorescence quantum yield of 0.60, demonstrating significant brightness compared to its highly quenched state in water where its quantum yield drops to 0.17. In contrast, 13 displays an absorption maximum at 497 nm and an emission maximum at 515 nm with a low fluorescence quantum yield of 0.03, which enhances significantly to 0.27 in highly viscous glycerol due to restricted intramolecular rotation. This structural design capitalizes on the distinct properties of each component: biotin provides high-affinity receptor recognition, whereas the self-quenched BODIPY dimer 14 ensures that fluorescence activation occurs predominantly upon membrane insertion, thereby minimizing background signal and enabling wash-free imaging. In bioimaging applications, the probe successfully discriminates biotin-receptor-overexpressing cancer cells from non-cancer cells when added at a low concentration of 200 nM, providing an immediate, high-contrast fluorescence signal at the plasma membrane within 2 minutes of incubation without requiring any washing steps. The wash-free nature of this system is particularly noteworthy because it addresses a major practical limitation of conventional fluorescent probes, namely background fluorescence arising from unbound dye molecules. Moreover, the fluorogenic strategy enables real-time visualization of receptor engagement while minimizing perturbation of cellular physiology.

**Fig. 6:**
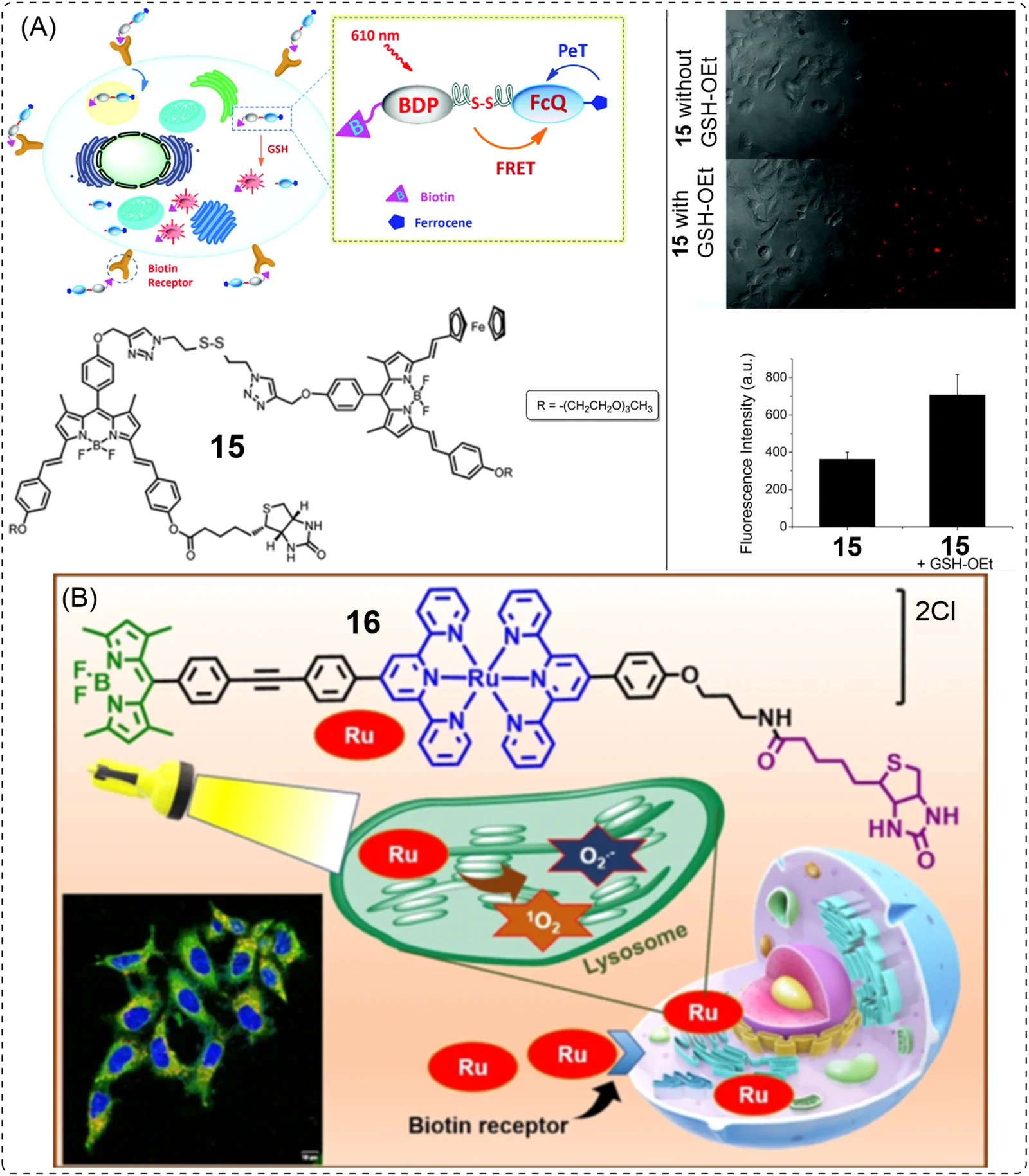
(A) Synthesis of the dual functional FRET-based fluorescent probe 15. Bright field and confocal fluorescence images of A549 cells after incubation with null or 10 mM GSH-OEt for 30 min, followed with 15 for 4 h (reproduced with permission.^69^ Copyright 2016, *Royal Society of Chemistry*). (B) Structure of dye 16 and schematic diagram of photodynamic antitumor effects (reproduced with permission.^70^ Copyright 2022, John Wiley & Sons).

Further sophistication has been achieved through the development of activatable probes that couple receptor targeting with intracellular biochemical sensing. In one example, a dual-functional molecular probe 15 was constructed by covalently conjugating the natural product biotin with a distyryl BODIPY fluorophore and a ferrocenyl styryl BODIPY quencher *via* carbodiimide coupling and copper(i)-catalyzed azide–alkyne cycloaddition, featuring a glutathione-cleavable disulfide linker (Fig. 6A).^69^ Evaluated in *N*,*N*-dimethylformamide (DMF), this fully assembled conjugate 15 exhibits a maximum electronic absorption wavelength of 638 nm with a molar absorption coefficient of 110 000 M^−1^ cm^−1^, a strong fluorescence emission maximum at 648 nm upon excitation at 610 nm, and a significantly diminished fluorescence intensity relative to its unquenched precursor with an *I*/*I*_0_ ratio of 0.20. Following intracellular thiol-mediated cleavage, fluorescence is restored selectively in biotin-receptor-positive cells. The probe 15 exhibits approximately 2.5-fold greater fluorescence intensity in human lung adenocarcinoma A549 cells than in receptor-negative controls and responds sensitively to elevated glutathione levels. Such activatable systems are conceptually attractive because they convert endogenous biomarkers into imaging contrast, thereby providing information not only on cellular localization but also on biochemical activity. This transition from static targeting to functional imaging represents an important evolution in the design of biotin-guided BODIPY probes.

Beyond purely organic photosensitizers and imaging probes, biotin has also been incorporated into increasingly sophisticated metal-containing theranostic platforms that combine multiple therapeutic modalities within a single molecular construct. The natural product biotin is covalently linked *via* an amide bond to an organic BODIPY chromophore, which is further coupled through a rigid diphenylacetylene spacer to a biocompatible terpyridyl ruthenium(ii) complex to form a complex, bichromophoric dyad architecture 16 (Fig. 6B).^70^ Evaluated in dimethyl sulfoxide (DMSO) solution, this integrated dyad 16 exhibits a maximum absorption wavelength of 503 nm, an exceptionally high molar absorption coefficient of 71 000 M^−1^ cm^−1^, a weak fluorescence emission maximum near 513 nm, and a significantly quenched fluorescence quantum yield of 0.003 relative to its isolated organic precursor due to efficient intramolecular energy transfer. This specific molecular configuration pairs the high-affinity biochemical targeting capacity of biotin toward overexpressed cell-surface receptors with the intense, light-harvesting property of the BODIPY component and the efficient intersystem crossing of the ruthenium core, establishing a complementary mechanism that channels the captured singlet-state energy into generating highly reactive triplet states rather than radiative emission. In photodynamic therapy (PDT) applications, 16 demonstrates outstanding cellular internalization and light-induced efficacy, generating a high singlet oxygen quantum yield of 0.63 in DMSO that translates into a remarkable, localized photocytotoxicity index against biotin-receptor-positive HeLa cervical cancer cells with a low half-maximal inhibitory concentration IC_50_ of 0.16 µM under visible light irradiation (400–700 nm, 10 J cm^−2^) while maintaining negligible toxicity in the dark (IC_50_ > 100 µM). The efficient energy transfer observed in this dyad demonstrates how transition-metal complexes can overcome one of the intrinsic limitations of many organic photosensitizers, namely inefficient triplet-state formation.

**Fig. 7:**
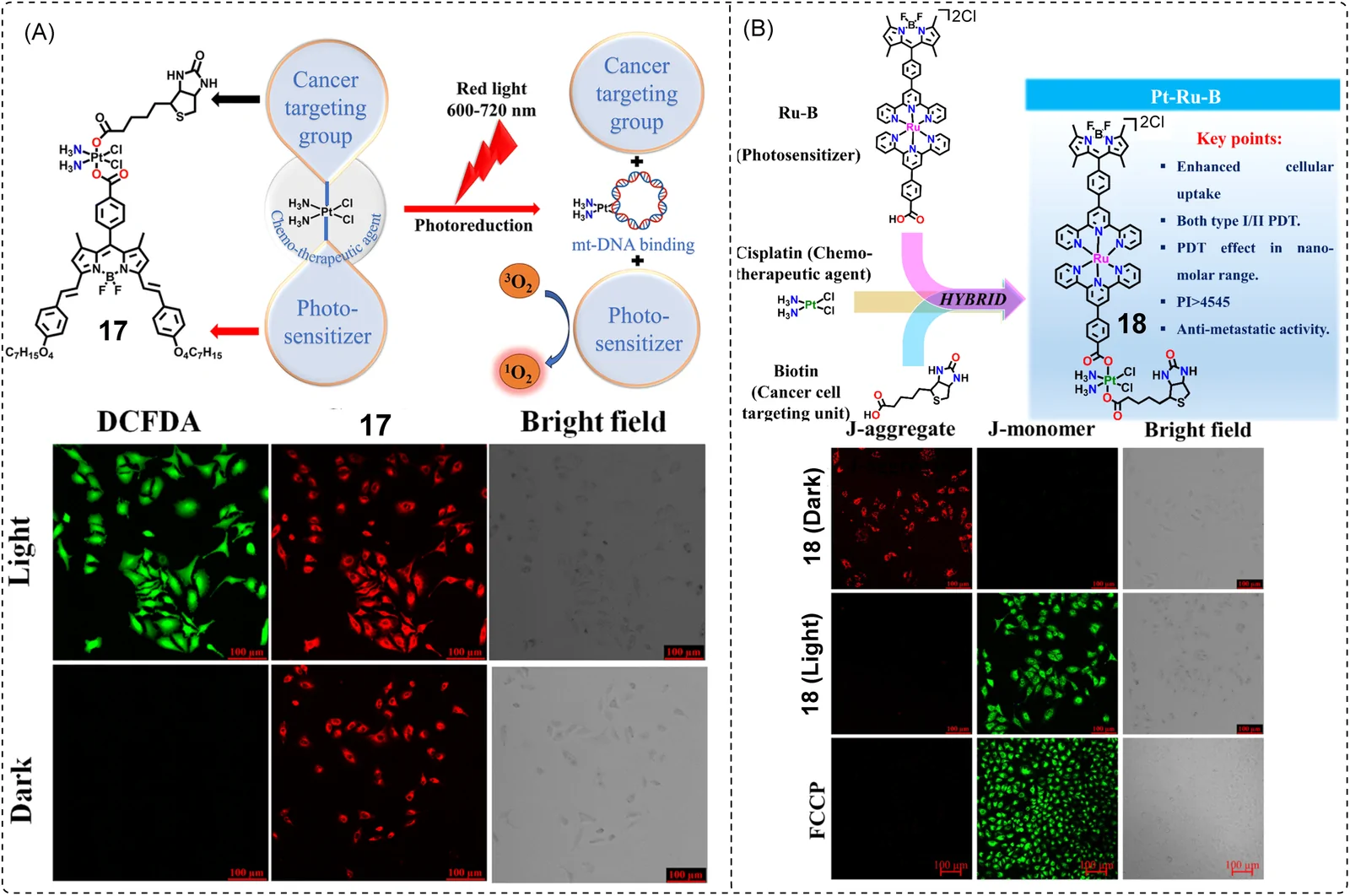
(A) Schematic representation of the chemical structure of the complex 17 and the concept behind the design of this prodrug generating chemo-active cisplatin and the photosensitizer with light activation. Confocal images of cells incubated with the IC_50_ concentration of complex 17 and DCFDA before and after light irradiation (reproduced with permission.^71^ Copyright 2023, *Royal Society of Chemistry*). (B) Chemical structure of the complex 18 and the concept behind the design of this prodrug. Pt–Ru–B generated chemo-active cisplatin and the photosensitizer upon reduction. Fluorescence images of the JC-1 dye assay at 10× magnification showing red and green fluorescence when A549 cells were incubated with 18 for 4 h at 37 °C. Carbonyl cyanide-4-(trifluoromethoxy) phenylhydrazone (FCCP) was used as a positive control (reproduced with permission.^72^ Copyright 2024, *American Chemical Society*).

One of the most straightforward applications of this concept is the construction of receptor-targeted photosensitizers. A representative example is the Pt(iv)–BODIPY–biotin prodrug *cis*, *cis*, *trans*-[Pt(NH_3_)_2_Cl_2_(biotin)(L)], in which a pseudo-octahedral Pt(iv) center simultaneously incorporates a biotin targeting ligand and a PEGylated BODIPY photosensitizer (Fig. 7A).^71^ The complex 17 displays a strong absorption band at 651 nm (*ε* = 9.19 × 10^4^ M^−1^ cm^−1^) and emits at 677 nm with a fluorescence quantum yield of 0.13. The unique architecture seamlessly merges the exceptional cancer-targeting specificity of biotin toward overexpressed cell transporters with the profound PDT performance of the BODIPY chromophore, achieving optimal synergy as heavy-metal coordination enhances the triplet state population to generate a high singlet oxygen quantum yield (0.32). In terms of biological applications, the prodrug 17 displays remarkable, selective photocytotoxicity against cancerous A549 lung cells with a low half-maximal inhibitory concentration IC_50_ of 0.61 ± 0.03 µM upon red-light irradiation, which represents an approximate 41-fold reduction in toxicity compared to non-cancerous human bronchial epithelial Beas-2B cells (24.51 ± 1.4 µM) while remaining completely non-toxic in the dark (IC_50_ > 100 µM). The large therapeutic window observed in this system highlights the value of combining receptor-mediated targeting with photoactivation, thereby introducing two independent layers of selectivity.

An even more elaborate example is provided by the heterobimetallic Pt(iv)–Ru(ii)–BODIPY prodrug, in which biotin serves as a targeting ligand while Pt(iv), Ru(ii), and BODIPY contribute complementary therapeutic functions. The biological oncotargeting natural product biotin is covalently bound as an axial ligand to a pseudo-octahedral Pt(iv) center, which is further linked to a Ru(ii)–BODIPY photosensitizer 18*via* a terpyridine-carboxylate bridge (Fig. 7B).^72^ This hybrid structure 18 possesses highly distinct photophysical properties, including a maximum electronic absorption wavelength of 500 nm, a high molar absorption coefficient of 4.43 × 10^4^ M^−1^ cm^−1^, a characteristically subdued fluorescence emission at 513 nm upon 488 nm excitation, and a heavily quenched fluorescence quantum yield below 0.01. The unique property of this 18 lies in the heavy-metal-induced enhancement of intersystem crossing which quenches the natural fluorescence of the BODIPY dye to drive outstanding type-I and type-II photodynamic therapy pathways, beautifully complementing the tumor-selective, carrier-mediated cellular uptake dictated by the structurally appended biotin. Its clinical potential is demonstrated by its remarkable submicromolar photocytotoxicity, achieving a half-maximum inhibitory concentration of approximately 0.022 µM in A549 lung cancer cells upon exposure to visible light while maintaining an exceptionally low toxicity in the dark IC_50_ > 100 µM, corresponding to a high phototoxicity index (PI) greater than 4545. From a molecular engineering perspective, this architecture represents a particularly sophisticated integration of three distinct functional modalities, receptor mediated recognition, metal based chemotherapeutic activity, and BODIPY sensitized photodynamic action, within a single covalent construct, demonstrating that careful spatial organization of these components can yield therapeutic indices far exceeding those of the individual agents.

Comparison across these biotin-based systems reveals an evolution toward increasingly sophisticated strategies for improving tumor targeting and therapeutic control. Early studies (*e.g.*, 9 and 10) primarily focused on receptor-directed delivery, exploiting biotin-associated transport pathways to enhance the accumulation of BODIPY chromophores in cancer cells. Subsequent designs incorporated stimuli-responsive elements, including pH-labile linkers (*e.g.*, 9), glutathione (GSH)-cleavable motifs (*e.g.*, 15), and photolabile groups, enabling spatially and temporally regulated activation of phototherapeutic functions. More recent approaches have further expanded the functional complexity of these systems through the incorporation of metal centers, such as Pt(iv) (17) and Ru(ii) (16 and 18), to integrate PDT with chemotherapy or photothermal therapy.

Notably, variations in phototoxicity indices (PI) among these systems appear to be associated with their photophysical properties, particularly their ability to generate reactive excited states. For example, compounds exhibiting efficient singlet-oxygen production, such as 16 (*Φ*_Δ_ = 0.63, PI > 66) and 18 (*Φ*_Δ_ = n.r.; exhibiting both Type I and Type II photochemical activity, PI > 4500), generally display enhanced phototherapeutic performance. In contrast, systems with lower ROS generation efficiency often show more limited therapeutic advantages. These observations suggest that, although biotin-mediated targeting contributes to selective cellular accumulation, optimization of BODIPY photophysical properties remains essential for maximizing therapeutic efficacy in targeted photodynamic platforms.

### 2.4 Capsaicin modified BODIPY

Capsaicin, a pungent vanilloid alkaloid derived from chili peppers, comprises a vanillyl aromatic ring connected *via* an amide bond to a hydrophobic alkyl chain, resulting in an amphiphilic molecular architecture that combines polar and lipophilic elements.^73^ Its principal molecular target is transient receptor potential vanilloid subtype 1 (TRPV1), a non-selective cation channel expressed not only in sensory neurons but also in a variety of malignant cells. Activation of TRPV1 by capsaicin induces Ca^2+^ influx, ROS accumulation, and mitochondrial dysfunction, ultimately leading to programmed cell death.^74^ Beyond this direct mechanism, capsaicin modulates multiple oncogenic signaling pathways, including NF-κB, PI3K/Akt, and STAT3, thereby suppressing tumor proliferation, invasion, and metastasis while promoting apoptosis. Anticancer activity has been reported against breast, prostate, colorectal, and liver cancers. Notably, capsaicin-induced oxidative stress can sensitize tumor cells to ROS-mediated therapies, suggesting an intrinsic compatibility with photodynamic treatment strategies. From a synthetic perspective, the phenolic hydroxyl and amide functionalities provide versatile sites for molecular modification. These characteristics make capsaicin particularly attractive for integration with BODIPY chromophores, as the natural product contributes well-defined biological activity and target engagement, whereas BODIPY offers precise optical control, fluorescence tracking, and photosensitization capabilities. Consequently, capsaicin–BODIPY systems have evolved along two complementary directions: photoactivatable molecular probes for spatiotemporal control of TRPV1 signaling and self-assembling theranostic conjugates designed to improve drug delivery and antitumor efficacy.

**Fig. 8:**
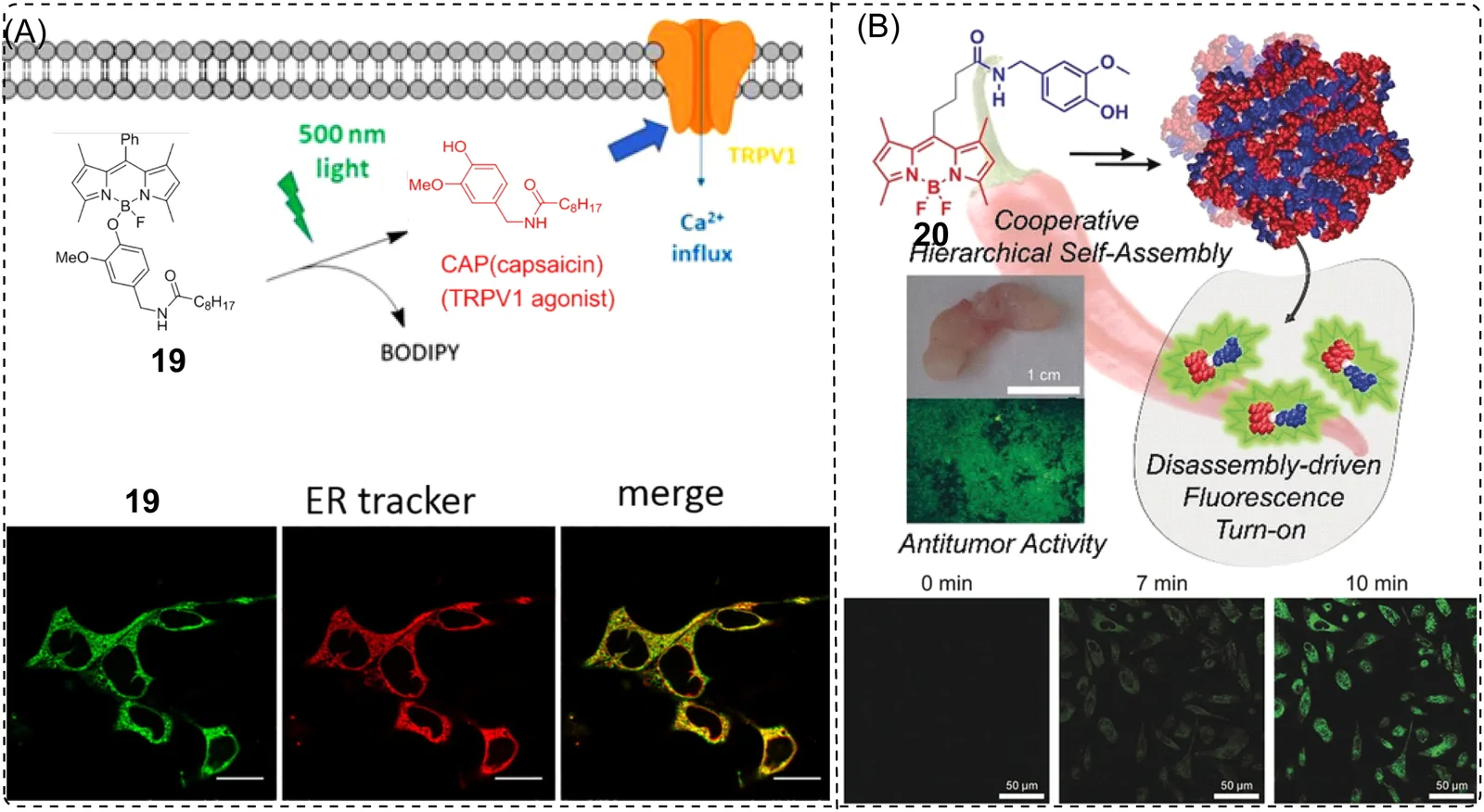
(A) Photoreaction scheme of 19. Intracellular localizations of 19 (green) and ER tracker (red), also colocalization of the two (yellow region) (reproduced with permission.^75^ Copyright 2018, Elsevier). (B) Chemical structure of 20, proposed hierarchical aqueous self-assembly and subsequent disassembly-driven emission turn-on upon cellular uptake (reproduced with permission.^76^ Copyright 2018, John Wiley & Sons).

One representative example is 19, which is formed by covalently linking the capsaicin analogue pelargonic acid vanillylamide (PAVA) to a BODIPY-based photolabile caging scaffold 19 through an aryloxy linkage, generating a π-conjugated donor–acceptor architecture capable of PeT-regulated uncaging (Fig. 8A).^75^ The conjugate 19 exhibits visible-light absorption near 500 nm with a molar absorptivity of approximately 6.4 × 10^4^ M^−1^ cm^−1^, fluorescence emission in the 510–525 nm region, a fluorescence quantum yield of approximately 0.01, and an uncaging quantum yield of 0.21 ± 0.03%. Structurally, the conjugate 19 combines the high extinction coefficient, membrane affinity, and traceable fluorescence of the BODIPY scaffold with the potent TRPV1-activating activity of capsaicin-derived PAVA, allowing optical regulation of biological activity while minimizing receptor activation prior to irradiation. Functionally, 19 exhibits an uncaging efficiency of 134 ± 19 M^−1^ cm^−1^, localizes efficiently within intracellular membranes including the endoplasmic reticulum, and induces rapid TRPV1-dependent Ca^2+^ influx in human embryonic kidney HEK293 cells following blue-green light irradiation. Importantly, this response is markedly suppressed by the TRPV1 antagonist capsazepine, confirming receptor-specific activation. This work establishes a general paradigm in which BODIPY-based photocages impart optical control over otherwise constitutively active natural products, enabling precise manipulation of TRPV1 signaling dynamics.

A distinct strategy utilizes the fluorophore as a supramolecular building block capable of modulating the pharmacokinetic behavior of capsaicin itself. This transition from molecular photopharmacology to self-assembled nanomedicine illustrates the versatility of BODIPY-based design in natural-product engineering. In this regard, the capsaicin–BODIPY conjugate (20) was generated through direct covalent attachment of capsaicin to a hydrophobic BODIPY fluorophore, affording an amphiphilic molecule capable of hierarchical self-assembly through π–π stacking, hydrogen-bonding interactions, and hydrophobic effects (Fig. 8B).^76^ The monomer 20 displays absorption maxima at 495–501 nm, whereas the aggregated species exhibits a red-shifted absorption band at 532 nm together with a secondary band at 468 nm. Fluorescence emission occurs at 503–509 nm in organic media, while aqueous aggregates exhibit weak red-shifted emission near 620 nm accompanied by substantial fluorescence quenching. Such aggregation-dependent optical behavior is particularly noteworthy because it provides direct spectroscopic evidence for supramolecular organization and may offer opportunities for aggregation-responsive imaging in biological environments. Structurally, capsaicin contributes intrinsic antitumor activity and hydrogen-bonding motifs that facilitate assembly, whereas BODIPY provides fluorescence functionality and drives formation of stable nanostructures. In biological studies, the self-assembled nanoclusters 20 exhibited diameters ranging from approximately 10–40 nm, with larger aggregates reaching up to 300 nm. The nanostructures accumulated efficiently in prostate tumors through the enhanced permeability and retention effect and significantly inhibited human prostate cancer PC-3 xenograft growth *in vivo* at a dose of only 18 mg kg^−1^. Remarkably, the effective dose required for antitumor activity was reduced by approximately two orders of magnitude relative to conventional capsaicin formulations while maintaining substantial therapeutic efficacy. This carrier free system is particularly notable not only for its dramatically reduced effective dose, approximately two orders of magnitude lower than conventional capsaicin formulations, but also for its inherent ability to integrate therapeutic action, nanostructure formation, and fluorescence tracking within a single molecular entity, thereby circumventing the need for auxiliary carrier materials.

The two capsaicin-based systems demonstrate how a common natural-product scaffold can be adapted toward distinct functional objectives through different molecular design strategies. The photocaged probe 19 serves as a chemical biology tool for spatiotemporal regulation of TRPV1 signaling, where the BODIPY unit acts primarily as a photoresponsive module while also enabling optical monitoring of the activation process. In contrast, the self-assembling conjugate 20 represents a therapeutic nanoplatform that exploits the hydrophobic and π-conjugated characteristics of BODIPY to promote carrier-free nanoassembly, resulting in a substantial reduction in the effective therapeutic dose. Together, these examples highlight the versatility of natural-product-BODIPY integration, demonstrating that rational molecular engineering can transform the same bioactive scaffold into either a mechanistic probe for biological interrogation or a multifunctional platform for therapeutic applications.

### 2.5 Paclitaxel modified BODIPY

Paclitaxel, a diterpenoid natural product isolated from *Taxus* species, represents one of the most successful examples of natural-product-derived anticancer therapeutics. Its structurally complex taxane framework, enriched with hydroxyl groups, ester functionalities, and the pharmacologically indispensable C13 side chain, exerts a unique mechanism of action by stabilizing microtubules against depolymerization, thereby inducing mitotic arrest at the G2/M phase and ultimately triggering apoptosis.^77,78^ Beyond direct cytotoxicity, paclitaxel influences multiple apoptotic pathways and modulates the tumor immune microenvironment. Despite its remarkable clinical success in the treatment of breast, ovarian, and non-small-cell lung cancers, its broader therapeutic utility remains constrained by poor aqueous solubility, dose-limiting systemic toxicity, and the emergence of multidrug resistance. From a synthetic perspective, the multiple hydroxyl functionalities provide versatile sites for selective derivatization. These structural features, particularly the multiple hydroxyl groups, make paclitaxel amenable to selective derivatization while largely preserving its microtubule-binding pharmacophore, thereby enabling the construction of theranostic conjugates with retained biological potency.

**Fig. 9:**
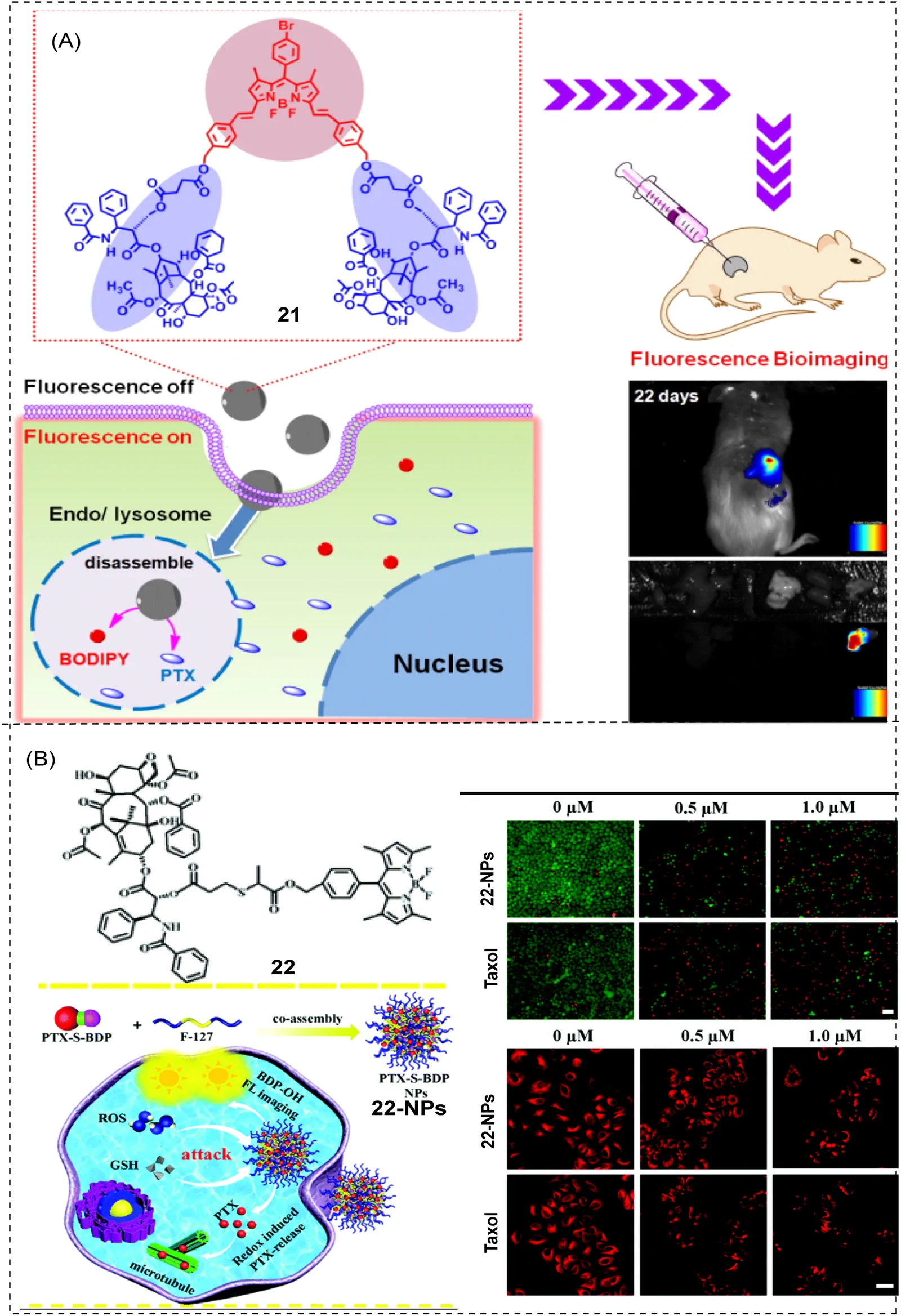
(A) Schematic illustration of the self-assembled nanoparticles from the small organic molecule 21, internalization and disassembly of the 21-NPs in the cells and their applications for NIR bio-imaging (reproduced with permission.^79^ Copyright 2018, Elsevier). (B) Structure of 22. Schematic illustration of the synthesis and controlled release of 22-NPs. Fluorescence images of HeLa cells co-stained with calcein-AM and PI after the treatment with 22-NPs or Taxol (reproduced with permission.^80^ Copyright 2021, *Royal Society of Chemistry*).

A representative example is conjugate 21, a symmetric brominated BODIPY-paclitaxel dimer in which two paclitaxel molecules are connected to a central BODIPY photosensitizer through enzyme-responsive ester linkages (Fig. 9A).^79^ The free conjugate 21 exhibits an absorption maximum at 632 nm, whereas self-assembled nanoparticles display a red-shifted absorption band at 639 nm together with fluorescence emission at approximately 688 nm. The fluorescence quantum yield of 21 is substantially reduced in the aggregated state owing to aggregation-caused quenching (ACQ). Although typically a drawback for imaging, fluorescence quenching here confirms dense molecular packing and may improve photostability during circulation. The carrier-free framework relies exclusively on intermolecular π–π stacking and hydrophobic interactions to generate stable nanostructures without the need for additional excipients or surfactants. Biologically, the nanoparticles of 21 exhibit an average diameter of 131.2 nm and a positive zeta potential of +21 mV, undergo rapid enzyme-responsive disassembly in the presence of proteinase K, and retain detectable near-infrared fluorescence at tumor sites for up to 22 days following administration.

Building upon the concept of self-reporting prodrugs, subsequent studies have focused on introducing chemically responsive linkers capable of coupling fluorescence behavior directly to drug activation. The redox-activated prodrug 22 was constructed by connecting paclitaxel to a BODIPY fluorophore through a monosulfide ester linker at the 2′-OH position of the paclitaxel scaffold (Fig. 9B).^80^ The conjugate exhibits characteristic BODIPY absorption near 500 nm and bright fluorescence emission in solution. Following nanoparticle formation with Pluronic F-127, the emission profile undergoes a pronounced red shift, reflecting changes in molecular packing and excited-state interactions. The 22-NPs possess an average hydrodynamic diameter of approximately 210 nm (PDI = 0.08). Importantly, the sulfur-containing linker functions not merely as a structural connector but as a molecular switch that couples the intracellular redox environment to prodrug activation. Such responsiveness is particularly relevant because elevated glutathione concentrations are a hallmark of many malignant tissues and can therefore provide a degree of tumor selectivity. The 22-NPs exhibit potent antiproliferative activity against HeLa cells, with an IC_50_ value of 0.02 µM after 72 h, while displaying substantially lower toxicity toward normal fibroblasts. Importantly, the fluorescence properties of the system are directly coupled to its activation state. Such self-reporting behavior transforms the fluorophore from a passive imaging tag into an active indicator of therapeutic activation.

**Fig. 10:**
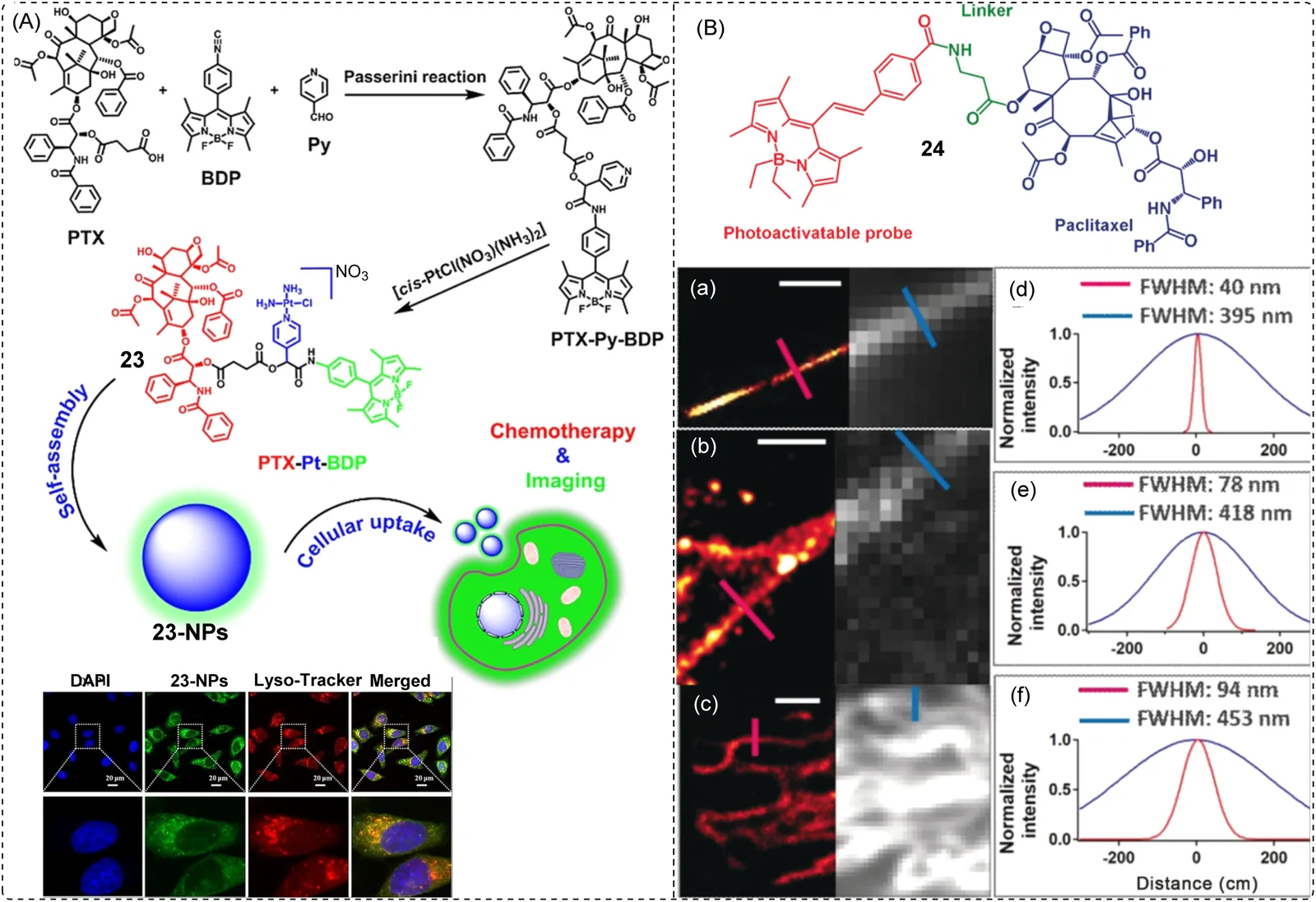
(A) Synthesis of 23 and its self-assembly into nanoparticles for cancer cell imaging and chemotherapy. CLSM images of A549 cells incubated with 23-NPs (5 µm) at 37 °C for 1 h. Pictures correspond to the fluorescence of DAPI (blue), 23-NPs (green), Lyso-Tracker red (red), and merged images from left to right (reproduced with permission.^81^ Copyright 2016, John Wiley & Sons). (B) Photoactivatable BODIPY probe 24 for SMLM imaging of microtubules. (a) *In vitro*, (b) fixed HeLa cell, and (c) live HeLa cell SMLM images of compound 24-labeled microtubules (color scale images). (d–f) Gaussian fits of line-scan intensities across the indicated areas in the SMLM images (red line) and corresponding diffraction limited images (blue line) of (a), (b), and (c), respectively (reproduced with permission.^82^ Copyright 2018, John Wiley & Sons).

Beyond prodrug activation, incorporation of additional therapeutic components offers opportunities to further expand functionality. An emerging strategy involves the construction of amphiphilic drug-dye conjugates capable of integrating chemotherapy, imaging, and metal-based therapy within a single molecular framework. The amphiphilic conjugate 23 was synthesized through a one-pot Passerini reaction linking paclitaxel and BODIPY, followed by coordination of a hydrophilic platinum-containing head group (Fig. 10A).^81^ Both the precursor and the metalized conjugate 23 exhibit absorption maxima at 501 nm, whereas self-assembled nanoparticles 23-NPs display broadened absorption and a 20 nm red-shift in fluorescence emission. Aggregation induces substantial fluorescence quenching, reducing the quantum yield from 0.54 to below 0.01. 23-NPs possess an average diameter of approximately 140 nm, a positive zeta potential of +49 mV, and remain colloidally stable for at least 15 days in aqueous media. Biologically, 23-NPs exhibit potent antiproliferative activity with IC_50_ values of 48 nM and 60 nM against A549 and MCF-7 cells, respectively, which are highly comparable to clinical Taxol while enabling real-time intracellular imaging and trafficking *via* confocal laser scanning microscopy. These results demonstrate that increasing molecular complexity does not necessarily compromise therapeutic potency when functionality is incorporated in a rational and complementary manner.

In contrast to the previous examples, which primarily focus on drug delivery and therapy, paclitaxel–BODIPY conjugates have also emerged as valuable tools for chemical biology and advanced microscopy. Because paclitaxel binds microtubules with exceptional specificity, it provides a highly attractive targeting vector for fluorescent probes capable of visualizing cytoskeletal dynamics. An illustrative example is a photoactivatable BODIPY–paclitaxel probe 24 in which paclitaxel is linked to a styryl-substituted boron-dialkylated BODIPY scaffold through an amide-containing linker (Fig. 10B).^82^ The conjugate 24 exhibits an absorption maximum at 498 nm, a molar absorptivity of 15 000 M^−1^ cm^−1^, fluorescence emission at 517 nm, and an exceptionally low initial fluorescence quantum yield of 0.002. Unlike conventional imaging probes that seek maximal brightness, this design intentionally suppresses fluorescence in the ground state to generate a low-background imaging environment. Such a strategy is particularly advantageous for super-resolution microscopy, where signal-to-noise ratio often dictates achievable spatial resolution. Upon green-light irradiation, the probe 24 undergoes boron-photodealkylation, resulting in approximately three-fold fluorescence enhancement. This photoactivation process of 24 enables single-molecule localization microscopy of microtubules with an average full-width-at-half-maximum diameter of 45 ± 10 nm under remarkably low laser irradiance. Paclitaxel functions not as a therapeutic cargo but as a molecular recognition element that directs the fluorophore to a highly specific intracellular target.

Across the paclitaxel-based conjugates, linker engineering represents a key design parameter for regulating drug release and therapeutic activation. Enzyme-responsive ester linkers (21) enable controlled paclitaxel liberation under tumor-associated conditions, whereas redox-responsive sulfur-containing linkers (22) facilitate activation within the intracellular glutathione-rich environment. The amphiphilic Pt(iv)–containing conjugate (23) further expands the functional scope of these systems by integrating fluorescence imaging with platinum-based chemotherapy and additional therapeutic modalities, although this multifunctionality is accompanied by increased structural complexity.

Notably, in compounds 21 and 22, the fluorescence response is closely coupled with the activation process, enabling self-reporting behavior in which the BODIPY fluorophore serves not only as an imaging component but also as an indicator of therapeutic release. This strategy highlights the potential of activatable fluorophore–drug conjugates for real-time monitoring of therapeutic processes and may provide a general design concept for other natural-product-based theranostic platforms. Such applications significantly broaden the scope of natural-product-BODIPY research by positioning paclitaxel not merely as a cytotoxic payload but as a molecular recognition element for high-resolution cytoskeletal imaging, thereby expanding the utility of these hybrids into the realm of chemical biology and super-resolution microscopy.

### 2.6 Doxorubicin modified BODIPY

Doxorubicin (DOX) is a clinically established anthracycline natural product originally derived from *Streptomyces* species. It remains one of the most widely used anticancer agents for the treatment of hematological malignancies and solid tumors, with its therapeutic activity primarily arising from DNA intercalation, topoisomerase II inhibition, and oxidative stress induction, leading to disruption of DNA replication and transcription.^83,84^ Despite its broad-spectrum efficacy, the clinical use of DOX is limited by dose-dependent cardiotoxicity, non-specific biodistribution, multidrug resistance, and systemic adverse effects. These limitations have motivated considerable efforts to integrate DOX with functional molecular platforms that can improve tumor selectivity, provide real-time imaging capability, and enable synergistic therapeutic mechanisms. Importantly, the evolution of DOX–BODIPY hybrids reflects a broader trend in nanomedicine. Early studies primarily focused on improving drug delivery and imaging capability. More recent designs increasingly integrate multiple therapeutic mechanisms, stimuli-responsive activation, supramolecular recognition, and self-reporting functionality. As a result, DOX–BODIPY systems now represent some of the most sophisticated examples of molecularly encoded theranostics.

**Fig. 11:**
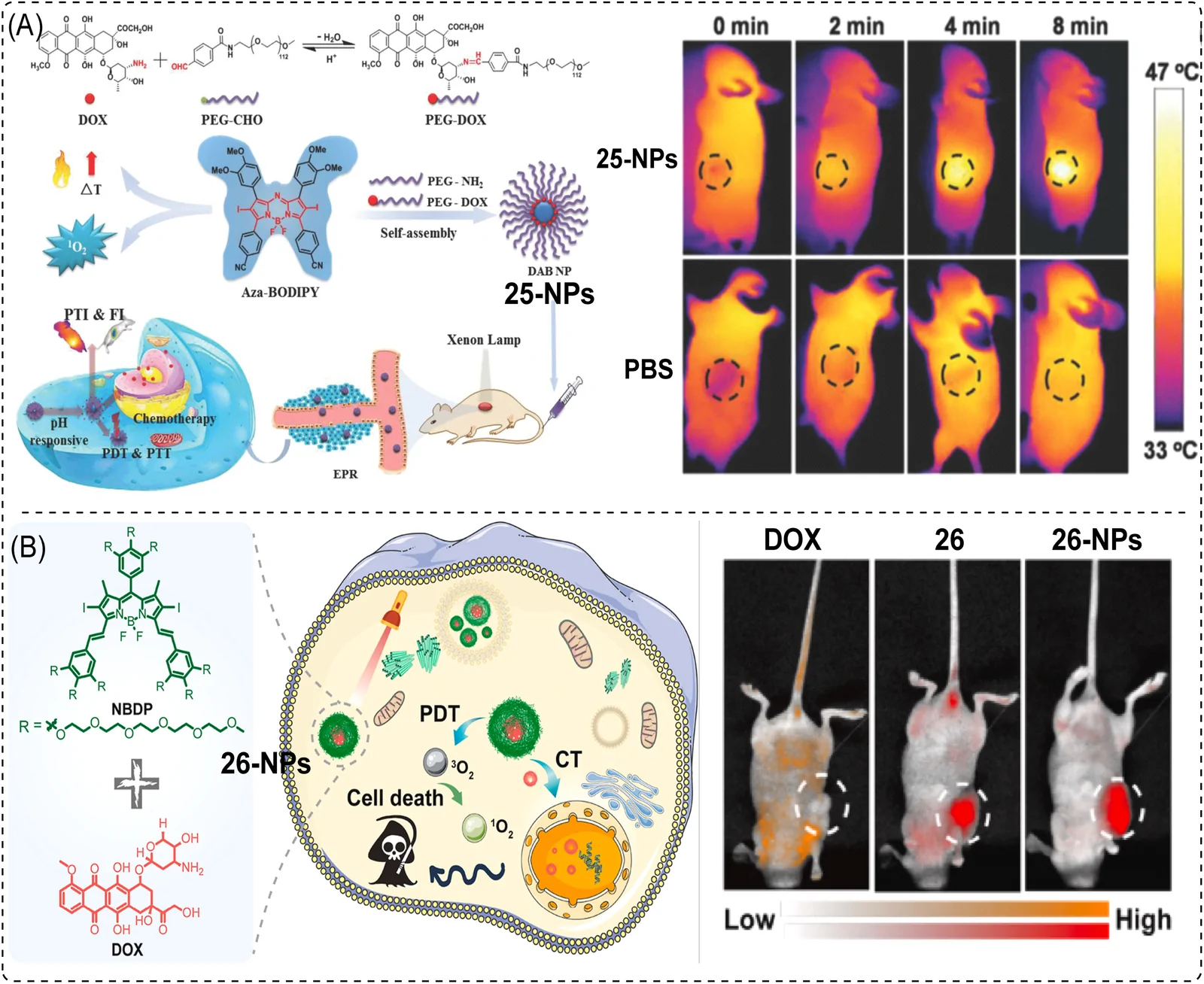
(A) Schematic illustration of the preparation of 25-NPs and their pH-responsive photodynamic/photothermal/chemo synergistic cancer therapy mechanism.^85^ Copyright 2018, John Wiley & Sons. (B) Schematic illustration of the synthetic process for 26/DOX NPs and the therapeutic performance of 26/DOX NPs. *In vivo* fluorescence tumour imaging (reproduced with permission.^86^ Copyright 2022, Elsevier).

To overcome the limitations associated with free doxorubicin while simultaneously exploiting the unique photophysical properties of BODIPY chromophores, one of the earliest and most widely explored approaches involves the incorporation of DOX into photoactive BODIPY-based nanoplatforms. The structural combination of the near-infrared photosensitizer Aza-BODIPY with the clinical natural product doxorubicin is achieved by non-covalent encapsulation within a hydrophilic polymeric matrix, where doxorubicin is pre-conjugated to polyethylene glycol *via* an acid-labile Schiff's base imine linkage (–HC=N–) (Fig. 11A).^85^ Photophysically, the resulting nanotheranostic agent 25-NPs exhibits distinctive electronic absorption maxima at 480 nm, corresponding to the doxorubicin component, and at 652 nm from the encapsulated Aza-BODIPY core; upon excitation, it generates a doxorubicin-centered fluorescence emission spanning 510 to 700 nm (peaking at 570 nm) along with near-infrared Aza-BODIPY dual emissions at 730 nm and 830 nm, supported by a high singlet oxygen quantum yield of 82% in dichloromethane. The coexistence of spectrally distinct chromophores is particularly advantageous because it permits simultaneous monitoring of carrier behavior and therapeutic cargo distribution while preserving both photodynamic and photothermal activities. From a structure–property perspective, the iodinated Aza-BODIPY core provides efficient intersystem crossing, high reactive oxygen species (ROS) generation, and photothermal conversion efficiency, whereas the acid-sensitive DOX conjugate introduces tumor-responsive drug release *via* cleavage of the Schiff's base HCN bond under acidic conditions, thereby creating a direct link between molecular design and therapeutic performance. This hybrid configuration of 25-NPs uniquely merges the therapeutic modalities of both entities, capitalizing on the DNA-intercalating chemotherapeutic toxicity of the anthraquinone-based natural product and the heavy-atom-facilitated intersystem crossing of the iodinated Aza-BODIPY core, which mutually compensate for single-agent limitations by preventing premature drug leakage in healthy tissues while maximizing localized destruction. Therapeutically, 25-NPs exhibit an exceptionally low half-maximal inhibitory concentration IC_50_ of 3.1 µg mL^−1^ against HeLa cells under broad-spectrum light irradiation, drive a cumulative pH-dependent doxorubicin release of up to 83.9% at an acidic pH of 5.0 within 48 hours, and achieve a photothermal conversion efficiency of 38.3% that elevates tumor site temperatures to 46.7 °C *in vivo*. Collectively, these features enable pH-responsive photodynamic/photothermal/chemo synergistic therapy, ensuring total tumor eradication after 12 days of treatment without recurrence.

Although nanocarrier-based systems provide important advantages, increasing formulation complexity often reduces translational feasibility. Consequently, recent efforts have shifted toward carrier-free nanomedicines in which the therapeutic components themselves participate directly in nanoparticle formation. A representative example is the self-assembled nanomedicine 26/DOX NPs (Fig. 11B).^86^ In this system, an amphiphilic iodinated BODIPY photosensitizer containing oligo(ethylene glycol) chains acts simultaneously as a therapeutic component and a structural assembly unit. Doxorubicin is subsequently incorporated through intermolecular interactions without the need for additional carrier materials. Photophysically, the uncomplexed amphiphilic 26 core displays a strong near-infrared electronic absorption peak at 660 nm in water and achieves an exceptional singlet oxygen quantum yield of 71.1% in *N*,*N*-dimethylformamide *via* heavy-atom-promoted intersystem crossing; upon loading the natural product, the resulting nanoparticles 26/DOX NPs exhibit enhanced absorption across the 350–600 nm range owing to the doxorubicin component, accompanied by a doxorubicin emission profile peaking at 556 and 592 nm along with a characteristic BODIPY emission peak (705 nm) in the near-infrared region. The design of 26/DOX NPs addresses a fundamental limitation of photodynamic therapy, namely its dependence on light penetration, by pairing a near infrared active iodinated BODIPY photosensitizer with doxorubicin. In this assembly, the BODIPY component provides spatially confined photodynamic activity, whereas the co-encapsulated doxorubicin ensures sustained DNA damaging cytotoxicity in tumor regions where light delivery or oxygen availability may be suboptimal, thereby achieving spatiotemporal complementarity between the two therapeutic actions. The BODIPY component provides spatially controllable photodynamic activity, whereas doxorubicin ensures sustained cytotoxicity in regions where light penetration and oxygen availability may be insufficient for efficient PDT. *In vitro* and *in vivo* evaluations demonstrate that these nanoparticles (26/DOX NPs) successfully leverage the enhanced permeability and retention effect to selectively accumulate in tumors, where under 660 nm laser irradiation (200 mW cm^−2^ for 6 min), they prompt intracellular reactive oxygen species generation that induces marked cytotoxicity in mouse mammary carcinoma 4T1 cancer cells, and deliver superior synergistic therapeutic efficacy in 4T1 tumor-bearing mice treated with a dosage of 9 mg per kg BODIPY to achieve near-complete tumor ablation after 14 days without inducing noticeable body weight loss.

**Fig. 12:**
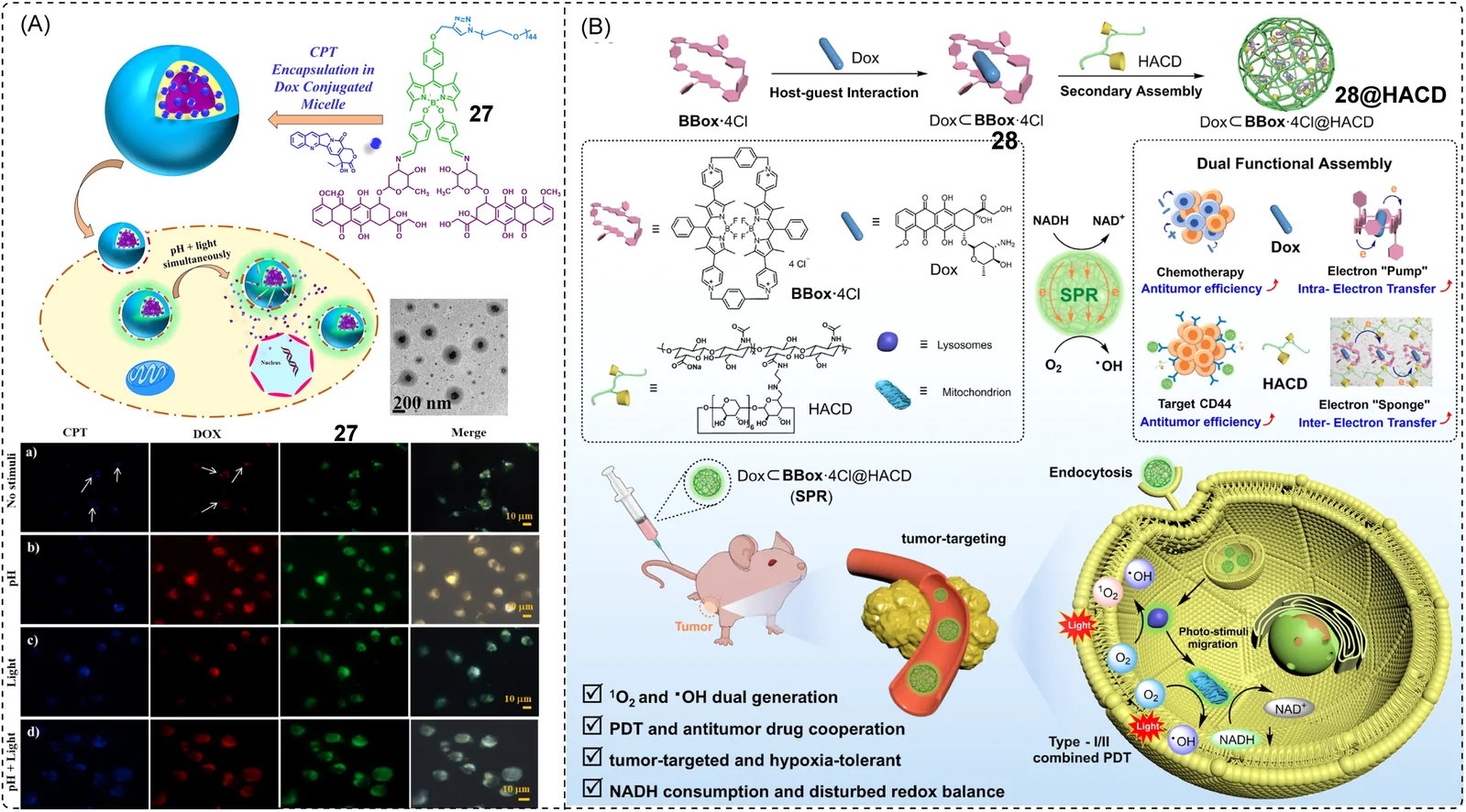
(A) Fluorescence images of MDA-MB-231 cells incubated with CPT loaded + Dox conjugated 27 micelles (reproduced with permission.^87^ Copyright 2024, Taylor & Francis) (B) Schematic illustration of the NADH-activated supramolecular photoelectron “reservoir” (SPR) for chemo-photodynamic synergistic tumor therapy of 28@HACD (reproduced with permission.^88^ Copyright 2025, *American Chemical Society*).

Covalent conjugation affords enhanced structural stability and precise stimulus-responsive drug activation. The structural combination of BODIPY and the natural product doxorubicin is achieved by covalent conjugation *via* acid-labile imine bonds (–HC=N–), which are formed through Schiff base condensation between an aldehyde group and the primary amine of doxorubicin *via* nucleophilic addition followed by dehydration, yielding an amphiphilic macromolecular prodrug architecture composed of a hydrophilic poly(ethylene glycol) chain linked through a central BODIPY core (Fig. 12A).^87^ Photophysically, the assembled macromolecular conjugate 27 exhibits a distinctive electronic absorption profile where the doxorubicin component is excited at 480 nm to yield a characteristic therapeutic fluorescence emission at 520 nm, which operates alongside the photoresponsive BODIPY core that selectively absorbs visible light at a maximum wavelength of 490 nm. Unlike the previously discussed self-assembled systems, the photophysical properties of this conjugate 27 are directly coupled to its chemical responsiveness, allowing optical excitation to participate in the regulation of drug release behavior. This hybrid arrangement exploits the unique complementarity of both components: the highly fluorescent BODIPY core incorporates visible light-cleavable phenoxy groups attached directly to the boron center to provide robust cellular tracking while triggering micellar disruption under green-blue light irradiation, whereas the conjugated anthracycline natural product introduces potent DNA-intercalating chemotherapy that releases autonomously within acidic microenvironments. *In vitro* evaluations on MDA-MB-231 breast cancer cells show that the blank nanocarrier is fully cytocompatible, whereas the doxorubicin-conjugated 27 micelles can physically encapsulate a second anticancer drug, camptothecin, with an exceptionally high encapsulation efficiency of 89% to facilitate simultaneous multi-drug delivery.

The most advanced generation of DOX–BODIPY architectures exploits supramolecular chemistry to achieve dynamic molecular recognition and cooperative therapeutic behavior. A representative example is the tetracationic BODIPY macrocycle 28, which forms a stable host–guest complex with doxorubicin through strong noncovalent interactions (Fig. 12B).^88^ Photophysically, the water-soluble 28 host exhibits a distinct visible light electronic absorption maximum at 511 nm with a shoulder at 496 nm in water, a narrow-band fluorescence emission centered between 541 and 547 nm, a high fluorescence quantum yield of 34.15% in acetonitrile that decreases to 5.34% in water owing to π-stacking aggregation, and an exceptionally high singlet oxygen quantum yield of 160%, whereas the addition of doxorubicin induces a further 5 nm bathochromic shift in the macrocycle's absorption profile. More importantly, the interaction between BODIPY and DOX extends beyond co-localization and contributes directly to the photochemical mechanism of action. This ternary supramolecular architecture leverages the unique, complementary properties of both entities: the electron-deficient tetracationic BODIPY macrocycle functions as a light-harvesting skeleton and a catalytic platform to capture electrons from intracellular nicotinamide adenine dinucleotide (NADH), while the encapsulated doxorubicin acts as an electronic “pump” that facilitates intramolecular photoelectron transfer between the twin BODIPY units, collectively creating a type-I/II combined photosensitization pathway that efficiently produces highly destructive hydroxyl radicals (˙OH) even under hypoxic conditions while preserving the drug's inherent DNA-intercalating toxicity. In terms of biological applications, the host–guest complex 28 undergoes secondary assembly with a hyaluronic acid derivative to form targeted nanoparticles that demonstrate a remarkable binding constant (*K*) of 8.05 × 10^5^ M^−1^, an impressive enhanced singlet oxygen quantum yield of 185% in the ternary system, and highly efficient tumor-targeted endocytosis that depletes intracellular NADH to disrupt redox homeostasis, culminating in a level of synergistic chemo-photodynamic efficacy against tumors that is markedly superior to either monotherapy, as evidenced by complete tumor regression without significant systemic toxicity *in vivo*.

The doxorubicin-based systems exemplify the diversification of strategies for integrating BODIPY photosensitizers with therapeutic cargos, ranging from carrier-assisted formulations to molecularly programmed architectures. In the early-stage design (*e.g.*, 25), polymeric nanocarriers were employed to co-deliver DOX and BODIPY components, providing efficient drug encapsulation while offering relatively limited control over molecular organization. Subsequent carrier-free approaches (26) utilized spontaneous self-assembly to construct defined nanostructures, thereby increasing drug content and minimizing potential toxicity associated with auxiliary excipients. Further advancement was achieved through covalent conjugation strategies (27), which enabled improved structural stability and stimulus-responsive drug release through precise molecular design. More recently, supramolecular host–guest architectures (28) have introduced dynamic molecular recognition and cooperative therapeutic functions, with intramolecular photoelectron transfer contributing to a 185% enhancement in singlet-oxygen quantum yield. Together, these examples demonstrate how increasingly precise control over molecular interactions and structural organization can enhance the performance of DOX–BODIPY theranostic systems. Rather than representing a simple chronological progression, these strategies provide complementary approaches for optimizing drug delivery, photodynamic activity, and functional integration.

## 3 Conclusions and future prospects

The studies in this review demonstrate that combining natural products with BODIPY fluorophores represents a versatile molecular engineering strategy spanning natural product chemistry, photochemistry, chemical biology, and nanomedicine. Natural products contribute biologically meaningful recognition, therapeutic activity, or self-assembly capability, whereas BODIPY provides optical functionality, environmental responsiveness, and phototherapeutic potential. Through rational integration of these complementary properties, researchers have generated a diverse range of molecular systems capable of imaging, sensing, drug delivery, photodynamic therapy, and multimodal theranostic applications. Several major design paradigms have emerged. (i) Mode of integration. Covalent conjugation offers precise control over stoichiometry and linker chemistry, making it the most widely applicable strategy. Coordination complexes exploit the electronic and catalytic properties of metal centers, enabling additional therapeutic mechanisms (*e.g.*, oxidative stress, chemotherapy). Supramolecular assemblies and nanoparticle formulations prioritize high drug loading and improved pharmacokinetics at the expense of molecular precision. The choice of integration strategy should therefore be guided by the intended application: molecular imaging and targeted therapy favor covalent conjugates, whereas systemic delivery benefits from nanoscale formulations. (ii) Stimuli-responsive systems couple optical activation to biochemical triggers such as pH, hypoxia, glutathione, enzymatic activity, or light exposure. Self-assembled carrier-free architectures exploit intrinsic physicochemical properties to achieve high drug loading and prolonged circulation. Multifunctional systems incorporating metal complexes or activatable motifs further integrate diagnosis, monitoring, and therapy. (iii) Natural-product selection. The choice of natural product dictates not only the biological activity but also the assembly behavior and subcellular localization of the hybrid. Cytotoxic natural products (paclitaxel, doxorubicin) contribute direct therapeutic effects and are suitable for chemotherapy-PDT combinations. Signaling modulators (capsaicin) enable spatiotemporal control of biological pathways and are ideal for chemical biology applications. Targeting ligands (biotin) improve tumor selectivity and are preferred for receptor-guided delivery. This diversity of natural-product functions underscores the versatility of the BODIPY platform and suggests that continued exploration of new natural-product classes will further expand the scope of the field.

Nevertheless, several challenges continue to limit the broader development and clinical translation of natural-product-BODIPY systems. First, inconsistent reporting of key photophysical parameters, including fluorescence quantum yields, triplet-state efficiencies, photostability, and excited-state properties, hampers direct comparison among different systems and limits the establishment of reliable structure–property relationships.^89,90^ More importantly, although many hybrids exhibit promising *in vitro* therapeutic effects, comprehensive pharmacokinetic (PK) and absorption, distribution, metabolism, and excretion (ADME) evaluations remain scarce. The biodistribution and biological fate of these systems are influenced not only by the targeting ligands but also by the lipophilic BODIPY framework and coordinated metal centers, which may affect protein binding, metabolism, and tissue accumulation. In addition, limited long-term toxicity studies, particularly for heavy-atom-containing photosensitizers and metal-based constructs, represent a critical barrier to translation.^91^ The increasing structural complexity of multifunctional theranostic systems also presents challenges in synthetic scalability, reproducibility, and manufacturing. Furthermore, regulatory evaluation remains challenging because these platforms combine features of therapeutic agents, imaging probes, and light-activated technologies. Finally, intrinsic limitations of PDT, including restricted light penetration, tumor hypoxia, and oxygen dependence, highlight the need for integrated delivery strategies and combination therapies beyond molecular optimization alone.^92^ Addressing these challenges will be essential for advancing these systems from proof-of-concept studies toward clinically relevant applications.

Looking forward, several emerging strategies may accelerate the development of next-generation natural-product-BODIPY platforms. Near-infrared and second near-infrared BODIPY derivatives may improve imaging depth and therapeutic applicability in deep tissues, while activatable systems responsive to multiple disease-associated signals could enhance selectivity and minimize off-target effects. Advances in aggregation-induced emission, photothermal conversion, and multimodal imaging may further overcome limitations associated with aggregation-caused quenching.^93,94^ In parallel, artificial intelligence-assisted molecular design, quantitative structure–property relationship analysis, and high-throughput screening provide new opportunities for optimizing photophysical properties, pharmacokinetic behavior, and synthetic accessibility.^95,96^ Future studies should prioritize standardized photophysical characterization, early-stage PK and toxicity assessment, and scalable synthetic strategies during molecular design. Ultimately, interdisciplinary integration of synthetic chemistry, photophysics, biology, and translational science will be crucial for transforming natural-product-BODIPY hybrids into practical tools for chemical biology and precision medicine.

## Conflicts of interest

The authors of this article declare no conflicts of interest.

## Abbreviations

BODIPY: Boron-dipyrromethene
DPBF: 1,3-Diphenylisobenzofuran
*Φ*_F_
: Fluorescence quantum yield
ISC: Intersystem crossing
ROS: Reactive oxygen species
ER-targeted: Endoplasmic reticulum-targeted
*Φ*_Δ_
: Singlet oxygen generation
IC_50_: Half maximal inhibitory concentration
HOMO: Highest occupied molecular orbital
LUMO: Lowest unoccupied molecular orbital
GA: Gambogic acid
COF: Covalent organic framework
SMVTs: Sodium-dependent multivitamin transporters
PeT: Photoinduced electron transfer
PEG: Poly(ethylene glycol)
BRs: Biotin receptors
FRET: Fluorescence resonance energy transfer
DMF: *N*,*N*-Dimethylformamide
DMSO: Dimethyl sulfoxide
PDT: Photodynamic therapy
DCFDA: DCFDA
*ε*
: Molar absorption coefficient
PI: Phototoxicity index
TRPV1: Transient receptor potential vanilloid subtype 1
PAVA: Pelargonic acid vanillylamide
ACQ: Aggregation-caused quenching
DAPI: 4′,6-Diamidino-2-phenylindole
DOX: Doxorubicin
NADH: Nicotinamide adenine dinucleotide
˙OH: Hydroxyl radicals
COFs: Covalent organic frameworks
NF-κB: Nuclear factor kappa-B
STAT3: Signal transducer and activator of transcription 3
PI3K/Akt/mTOR: Phosphoinositide 3-kinase/protein kinase B/mammalian target of rapamycin
MAPK: Mitogen-activated protein kinase
ER: Endoplasmic reticulum
MCF-7: Michigan cancer foundation-7
TfR1: Transferrin receptor 1
Hsp90: Heat shock protein 90
NF-κB: Nuclear factor kappa-B
VEGFR2: Vascular endothelial growth factor receptor 2
Aza-BODIPY: Aza-boron-dipyrromethene
GSH: Glutathione
PK: Pharmacokinetic
ADME: Absorption, distribution, metabolism, and excretion

## Data Availability

No new data were generated or analysed as part of this review.
